# Large Variations in Phenylalanine Concentrations Associate Adverse Cardiac Remodelling in Adult Patients With Phenylketonuria—A Long‐Term CMR Study

**DOI:** 10.1002/jcsm.13667

**Published:** 2025-01-10

**Authors:** Radu Tanacli, Patrick Doeblin, Alessandro Faragli, Jan‐Hendrik Hassel, Christian Stehning, Ursula Plöckinger, Athanasia Ziagaki, Sebastian Kelle

**Affiliations:** ^1^ Department of Cardiology Angiology and Intensive Care Medicine, Deutsches Herzzentrum der Charité Berlin Germany; ^2^ Charité‐Universitätsmedizin Berlin, Corporate Member of Freie Universität Berlin and Humboldt‐Universität Zu Berlin Berlin Germany; ^3^ Philips Clinical Science Hamburg Germany; ^4^ Interdisziplinäres Stoffwechsel‐Centrum Charité‐Universitätsmedizin Berlin, Campus Virchow Klinikum Berlin Germany; ^5^ Department of Endocrinology, Diabetes, and Nutrition Charité University Medicine Berlin Berlin Germany; ^6^ German Centre for Cardiovascular Research DZHK, Partner Site Berlin Berlin Germany

**Keywords:** cardiac magnetic resonance imaging, cardiac remodelling, contractile impairment, metabolic disease, phenylketonuria

## Abstract

**Background:**

Despite a phenylalanine (Phe) restrictive diet, most adult patients with ‘classical’ phenylketonuria (PKU) maintain life‐long Phe concentrations above the normal range and receive tyrosine (Tyr) and protein‐enriched diets to maintain acceptable concentrations and ensure normal development. While these interventions are highly successful in preventing adverse neuropsychiatric complications, their long‐ term consequences are incompletely explored. We observed early cardiomyopathic characteristics and associated hemodynamic changes in adult PKU patients and present here the results of a longitudinal evaluation of cardiac phenotype.

**Methods:**

Fifteen adult patients with PKU (age: 39.8 ± 8.1 years, 9 males and 6 females) underwent a comprehensive follow‐up cardiac magnetic resonance (CMR) imaging assessment after a mean follow‐up interval of 8.3 ± 0.3 years from the initial baseline visit. The CMR protocol included left (LV) and right (RV) ventricular and left atrial (LA) volumetric assessment, LV parametric mapping (precontrast and postcontrast T1 and T2 maps, extracellular volume [ECV]), multilayer LV myocardial strain, systolic and diastolic hemodynamic forces and RV and LA strain and aortic distensibility evaluation. Plasma concentrations of Phe, tyrosine (Tyr) and other biochemical markers of disease were retrospectively collected. For comparison, a group of 20 matched control subjects undergoing an identical CMR protocol was included.

**Results:**

On average, the LV end‐diastolic volume (EDV) (158 ± 29 vs. 143 ± 29 mL, *p* = 0.013) and end‐systolic volume (ESV) (68 ± 18 vs. 62 ± 18 mL, *p* = 0.011) were lower at follow‐up. In contrast, LV mass (LVM) (72 ± 25 vs. 82 ± 29 g, *p* < 0.001) and the ratio LVM/EDV (0.46 ± 0.12 vs. 0.58 ± 0.23 g/mL, *p* = 0.005) were increased, and T1 times were longer (940 ± 42 vs. 1010 ± 35 ms, *p* < 0.001). LV EF (57 ± 6 vs. 57 ± 7%, *p* = 0.90), longitudinal (GLS) and circumferential (GCS) systolic strain remained unchanged, but early diastolic hemodynamic (HD) forces were more markedly negative (−19.4 ± 7.0 vs. −26.5 ± 12.2%, *p* = 0.012), while LA strain 43.8 ± 11.3 vs. 37.3 ± 9.6%, *p* = 0.031) and aortic distensibility (6.38 ± 1.75 vs. 5.21 ± 1.17 10^−3^ mmHg^−1^, *p* = 0.008) decreased at follow‐up. Compared with controls, PKU patients maintain reduced systolic function with lower LV EF and impaired GCS and have more markedly negative early diastolic HD pressures. A higher decrease in Phe concentration (ΔPhe) was associated with longer T1 times, ΔT1 (*β* = −0.78, *p* < 0.001), increased ECV, ΔECV (*β* = −0.61, *p* = 0.016) and a decrease in systolic function, ΔEF (*β* = 0.61, *p* = 0.017). In contrast, variations in Tyr concentrations did not affect the cardiac phenotype.

**Conclusions:**

At long‐term follow‐up, a marked drop in Phe plasma concentration was associated with detrimental cardiac remodelling consisting of decreased LV systolic function and increased diffuse fibrosis, in PKU patients. These new data prompt further investigation into the effects of large Phe variability over time and underline the usefulness of periodic cardiovascular assessment in adults with PKU.

**Abbreviations**
2Ch, 3Ch, 4Ch: 2 chamber, 3 chamber, 4 chamber view
Ao: aorta (aortic)
CI: cardiac index
CMR: cardiac magnetic resonance
CO: cardiac output
E: trans‐mitral flow velocity
e’: early diastolic tissue velocity
ECV: extracellular volume
EDV: end‐diastolic volume
Endo: subendocardial layer
ESV: end‐systolic volume
GCS: global circumferential strain
GLS: global longitudinal strain
GLM: general linear model
HD: hemodynamic
HDL: high‐density lipoproteins cholesterol
HF: heart failure
LA, RA: left/right atrium (atrial)
LDL: low‐density lipoproteins cholesterol
LV, RV: left/right ventricle (ventricular)
LVM: left ventricular mass
MOLLI: modified Look‐Locker Inversion recovery
Myo: midmyocardial layer
PAH: phenylalanine hydroxylase
Phe: phenylalanine
PKU: phenylketonuria
SENSE: sensitivity encoding
SSFP: steady‐state free precession imaging
SV: stroke volume
TE: echo time
TR: repetition time
Tyr: tyrosine

## 1 Introduction

Phenylketonuria (PKU) is an autosomal recessive Mendelian disease affecting amino acid metabolism, characterised by the complete absence of phenylalanine (Phe) hydroxylase expression. This deficiency leads to impaired catabolism and toxic accumulation of Phe, resulting in systemic and organ complications [1]. Early diagnosis at birth and strict adherence to a Phe‐restricted diet, avoiding foods such as meat, fish, cheese, nuts and other protein‐rich nutrients [2], have proven highly effective in ensuring normal development and preventing neurological deficits during childhood (Supporting Information S1). To maintain metabolic balance and prevent complications in adulthood, PKU patients are advised to continue limiting Phe intake to 25% of normal levels. Regular monitoring of Phe concentrations through clinical evaluations and dietary consultations is recommended. Current clinical guidelines [2, 3] recommend a Phe concentration within a generic target area [4], but clinical practice regarding an optimal range of Phe and vigilance in checking these concentrations into adult life vary significantly among different European countries and the United States [5, 6] (Supporting Information S2). While multicentric clinical data demonstrate the efficacy of these measures in preventing neurological damage, ensuring normal development and maintaining cognitive and social functions [7], recent findings using brain magnetic resonance imaging reveal subtle white matter changes, demyelination and persistent inflammation in adult PKU patients, even with mean Phe levels as low as 600 μmol/L, a value below current guideline thresholds. These neurological changes appear more severe in older patients with chronically elevated Phe levels [8].

Adult PKU patients also face risks such as abnormal metabolite accumulation, increased oxidative stress and impaired protein and cholesterol synthesis, which persist throughout life [9]. The long‐term effects of elevated Phe levels on organs such as the heart and vasculature remain insufficiently explored. We found previously several incipient cardiac remodelling traits in adult PKU patients, particularly low T1 times, decreased LV (left ventricular) mass with thin LV walls, and we related these changes to higher Phe and lower tyrosine (Tyr) plasma levels [10]. Functionally, PKU patients exhibit lower average LV ejection fraction (EF) and impaired global circumferential stress (GCS), along with higher diastolic HD stress (more markedly negative HD forces) compared to healthy subjects. Furthermore, these differences are more pronounced in a subgroup of PKU patients with overt dyslipidemia. Several lines of evidence support the notion that these initial changes observe in relatively young adults may be progressive in nature and therefore monitoring changes in their cardiac phenotype may be informative for early detection and targeted treatment. Cardiac phenotype characterised by enlarged end‐diastolic volume (EDV) and eccentric remodelling, as the one observed in PKU patients, relying on increased Frank‐Starling forces to maintain physiological cardiac output, is maladaptive and leads in time to functional decompensation [11]. Conversely, chronically elevated levels of Phe were previously associated with a higher incidence of heart failure (HF) in general population [12], likely through higher levels of oxidative stress (Supporting Information S4) and interference with cellular metabolism, leading to impaired oxidative phosphorylation and ATP production [9]. Harbouring higher levels of reactive oxygen species and lower antioxidant defences, adult PKU patients have a significant degree of endothelial dysfunction, proportional to the levels of plasma markers of oxidative stress [13]. Additionaly, many adult PKU patients tend to have a higher body mass index (BMI) [14] and some degree of sarcopenia [15] compared with healthy individuals. This combination likely exacerbates decompensation through an imbalance between increased metabolic demands, higher afterload and reduced myocardial mass and contractile reserve [16]. While a higher BMI, frequently observed in PKU patients, could independently contribute to adverse cardiac remodelling and impaired diastolic and systolic functions [17], the prevalence of clinical obesity (BMI ≥ 30 kg/m2) among adult PKU patients remains a topic of debate [18].

Therefore, since data on the progression of cardiac remodelling in PKU patients are currently lacking, we consider it crucial to longitudinally assess these changes over an extended period using gold‐standard tools such as cardiac magnetic resonance (CMR) imaging. The objectives of this study are twofold: (1) to evaluate the potential progression of pathological cardiac modifications observed initially in adult PKU patients and compare this phenotype with that of a matched healthy population and (2) to investigate any correlation between the biochemical manifestation of the disease (quantified by Phe plasma concentrations) and these definitive cardiac parameters.

## 2 Methods

### 2.1 Study Population

The study was approved by the Ethics Committee of the Charité Universitätsmedizin Berlin, complied with the Declaration of Helsinki and was registered at the German Register for Clinical Studies (DRKS00001120). All PKU patients included in the baseline study received an invitation to participate for the follow‐up study from the Interdisciplinary Center for Metabolism: Endocrinology, Diabetes and Metabolism at the Charité, University‐Medicine, Berlin, in agreement with the local Ethics Committee approval. The study content and purposes were explained in full to all invited patients personally and via a patient information leaflet or via telephone. Inclusion criteria were similar at the baseline recruitment and follow‐up: All patients were diagnosed at birth with “classical” PKU, were on a specific Phe‐restricted diet and had a satisfactory record of regular medical follow‐ups. Laboratory data, including repeated values of Phe and Tyr (usually obtained once per each term), were obtained from clinical registry of Charité Universitätsmedizin Berlin, upon obtaining an in‐person or telephone consent.

From the entire baseline cohort of 39, 15 patients were invited and agreed to participate in the CMR follow‐up study. Exclusion criteria were generic contraindications to CMR (claustrophobia, significant hearing impairment, morbid obesity, presence of metallic implants, artificial valve or cerebral aneurysm clips) or other significant cardiovascular pathology (uncontrolled arterial hypertension, coronary artery disease or history of unstable angina or myocardial infarct, history of acute or chronic myocarditis, recent diagnostic of clinically significant COVID‐19, hemodynamically relevant valve disease, arrhythmia).

Quantification of fat mass, fat‐free mass, visceral fat, basal metabolic rate, muscle mass, body water was collected retrospectively from the bioelectrical impedance analysis chart, performed routinely for each PKU patient during the clinical visits.

### 2.2 CMR

The 15 patients included in this had another, follow‐up, CMR scan with the same image acquisition and image analysis protocol as at baseline. For comparison, a group of 20 age and sex‐matched control subjects, undergoing a similar protocol of image acquisition and analysis, were randomly and retrospectively selected from our local database at the German Heart Centre of Charite (DHZC). Image acquisition protocols were in full compliance with the recommendations of the Society for Cardiovascular Magnetic Resonance (SCMR) [19]. All the CMR images were acquired on a 1.5 T (Achieva, Philips Healthcare, Best, The Netherlands) clinical system. From localisers, ECG‐gated balanced steady‐state free precession (bSSFP) sequences with 30 frames per cardiac cycle were used as follows: 3 long‐axis cine images corresponding to the standard 2 chamber (Ch), 3Ch and 4Ch views and a stack of short axis views from the plane of the mitral valve to the cardiac apex covering the entire LV and right ventricular (RV) volume. All these images were acquired under breath‐holds at end‐expiration, using the following imaging parameters: repetition time (TR) = 3.2 ms, echo time (TE) = 1.6 ms, flip angle = 60°, voxel size = 1.8 × 1.7 × 8.0 mm^3^. As contrast substance, 0.15 mmol/kg of gadobutrol/Gd‐DO3A‐butrol (Gadovist© produced by Bayer Vital GmbH Deutschland) was used. For late gadolinium enhancement (LGE) imaging, we used a T1‐weighted phase‐sensitive inversion recovery sequence in multiple orientations covering the three long axes and an entire short‐axis stack. Native and 15‐min postcontrast T1‐maps were performed using a modified Look‐Locker Look‐Locker Inversion recovery (MOLLI) sequence in a single midventricular short‐axis slice. Acquisition parameters followed the standard protocol established in our clinic: acquired voxel size = 2.0 × 2.0 × 10 mm^3^, reconstructed voxel size = 0.5 × 0.5 × 10 mm^3^, balanced SSFP readout, flip angle = 35°, parallel imaging (SENSE) factor = 2 and a 5s(3s)3s acquisition scheme.

### 2.3 Image Analysis

**FIGURE 1:**
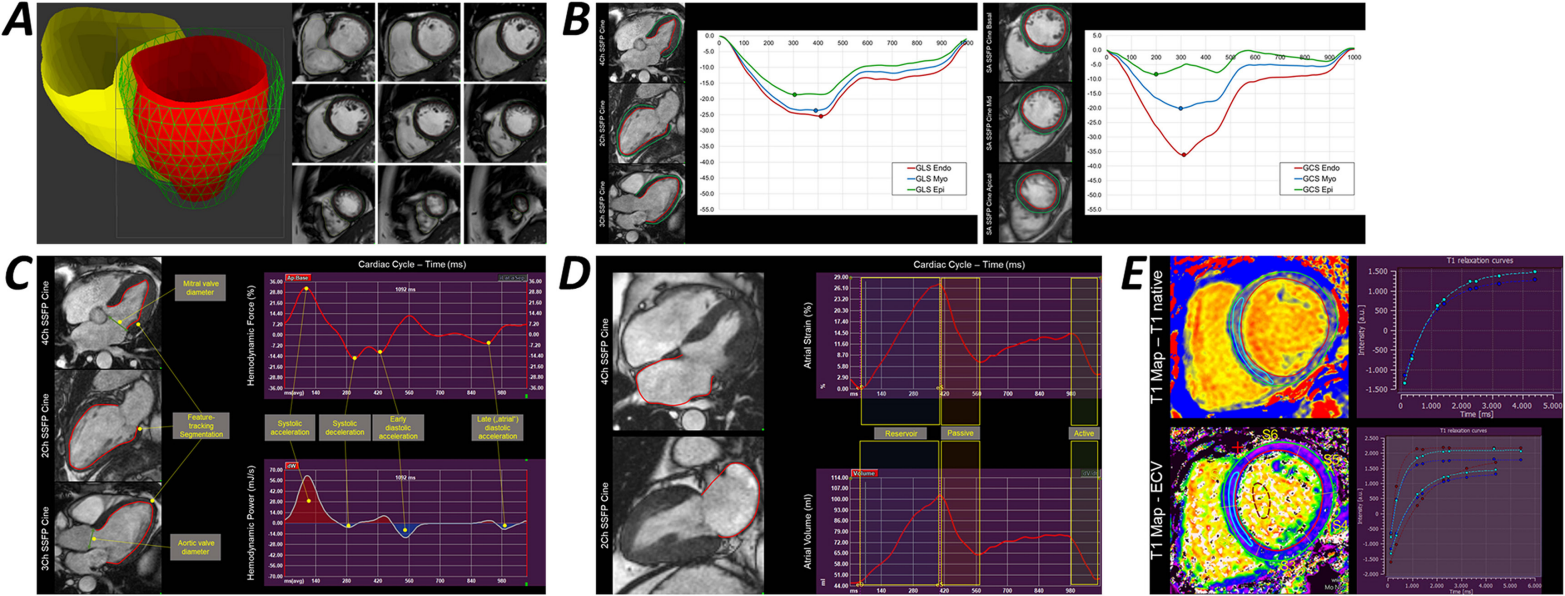
CMR methods used to characterise the cardiac phenotype of PKU patients: (A) LV and RV volumes were determined applying Simpson's approximation to multiple short‐axis SSFP cine images covering the ventricles from base to apex. (B) LV and RV myocardial longitudinal (GLS) and circumferential (GCS) strain was derived at multilayer level using a CMR feature‐tracking integrated algorithm, (C) Left ventricular hemodynamic (HD) forces corresponding to four phases of the cardiac cycle: systolic acceleration corresponding to the systolic thrust, systolic deceleration—recoil phase, early diastolic acceleration (suction), late (atrial) diastolic deceleration. (D) CMR feature tracking to assess atrial phasic function and strain. (E) parametric maps: T1 native and extracellular volume (ECV) colour‐encoded maps offer in combination information about structural changes at the myocardial level and assess their degree with a colour‐encoded quantitative scale.

All images were segmented and analysed using a commercially available software package (Medis Suite version 3.1, Leiden, The Netherlands) in line with the expert consensus and recommendations published by the SCMR. The short axis stack was segmented to calculate LV and RV volumes, LV and RV EFs and LV mass. Septal and lateral maximal LV wall thickness and LV maximal diameter were measured in end‐diastole at the level of the LV base in short‐axis slices. LV global longitudinal strain (GLS) and GCS were assessed at 2 distinct levels of the LV myocardium: Endo corresponding to the subendocardial region and Myo corresponding to the midmyocardium, using a feature‐tracking approach provided by QStrain v2.0 (Medis Suite) as described elsewhere [20]. Similarly, RV strain was assessed by contouring the endocardial contour and using feature‐tracking. Using mathematical modelling of myocardial deformation and volume curves over the cardiac cycle and, respectively, mitral and aortic valves diameters, we previously demonstrated the feasibility and clinical importance of deriving LV systolic, early diastolic and late diastolic hemodynamic (HD) forces, work and power as previously described [20]. We applied here an in‐built image processing tool (QStrain v2.0, Medis Suite) to derive these novel parameters for the PKU patients included in this study. Left atrial (LA) maximal and minimal volumes and global emptying fraction were assessed by contouring at maximal and minimal volume phases the LA circumference in 4‐Chamber (4Ch) and 2‐Chamber (2Ch) Steady‐State Free Precession (SSFP) cine and further calculated applying biplane area‐length method. LA strain was measured applying feature‐tracking to LA circumference in 2Ch and 4Ch respectively, and subsequently, averaging these values. Aortic distensibility was calculated segmenting the minimal and maximal transversal diameters of the descending aorta as identified in the bSSFP cine 4Ch view and indexing these values with simultaneously measured blood pressure values, as validated previously [21]. Native and post contrast T1 relaxation times were calculated using an operator defined region of interest in the LV septum at midventricular level. Haematocrit values were acquired immediately before the CMR scan using a point of care analysis and used in the previously described formula to calculate the extracellular volume (ECV) [22]. A summary of the CMR image acquisition methods and analysis is presented in Figure 1.

### 2.4 Biochemistry

Phe and Tyr concentrations were measured in dried blood spots using mass spectrometry. Phe and Tyr plasma concentrations were collected extensively covering the entire period between the baseline and follow‐up visits (between 4 and 10 determinations per year were available for each patient). As Phe concentrations may fluctuate between several visits, in order to give an accurate and more clinically relevant account, we averaged the Phe and Tyr concentrations over a period of 2 years prior to each CMR visit (baseline and follow‐up). These data were all obtained from the local database of the Interdisciplinary Center for Metabolism (Interdisziplinäres Stoffwechsel‐Centrum, SWC) of the Charité‐Universitätsmedizin Berlin. In addition, the most recent serum levels of total cholesterol, low‐density lipoprotein (LDL) cholesterol, high‐density lipoprotein (HDL) cholesterol, triglycerides, free fraction and total carnitine and glucose were obtained in the laboratory of the SWC. All routine determinations were performed in duplicate by the Hospital laboratory as part of the routine clinical evaluation of the patients (Labor Berlin‐Charité Vivantes GmbH).

### 2.5 Statistical Analysis

Normality of distribution for continuous variables was assessed by visual assessment of normality curves and the Shapiro–Wilk test. Where a variable failed to satisfy the normality distribution condition, log transformation or nonparametric equivalent tests were used. Comparisons between groups for continuous variables were performed with a two‐sided, paired‐samples Student's *t*‐test for normally distributed data and a paired‐samples Wilcoxon test for skewed data. When multiple comparisons were applied to the same set of variables, Bonferroni corrections were made. For categorical variables, comparison was done with a χ2 test and statistical significance evaluated with Fisher's exact test. Results are presented as mean **±** standard deviation (SD). Values of *p* < 0.05 were considered statistically significant. To account for interaction between different predictors (categorical and/or continuous variables), a general linear model was used. Univariate linear regressions were used to establish the correlation between variation of Phe and Tyr and, respectively, variation in other parameters characterizing clinical status or cardiac remodelling. To account for the co‐variate effect of age and BMI on these linear regression, multivariate linear regression models with age and BMI as co‐variates were used. All statistical tests were performed with R, version 4.4.1 (R Foundation for Statistical Computing, Vienna, Austria).

## 3 Results

### 3.1 Population Characteristics

**FIGURE 2:**
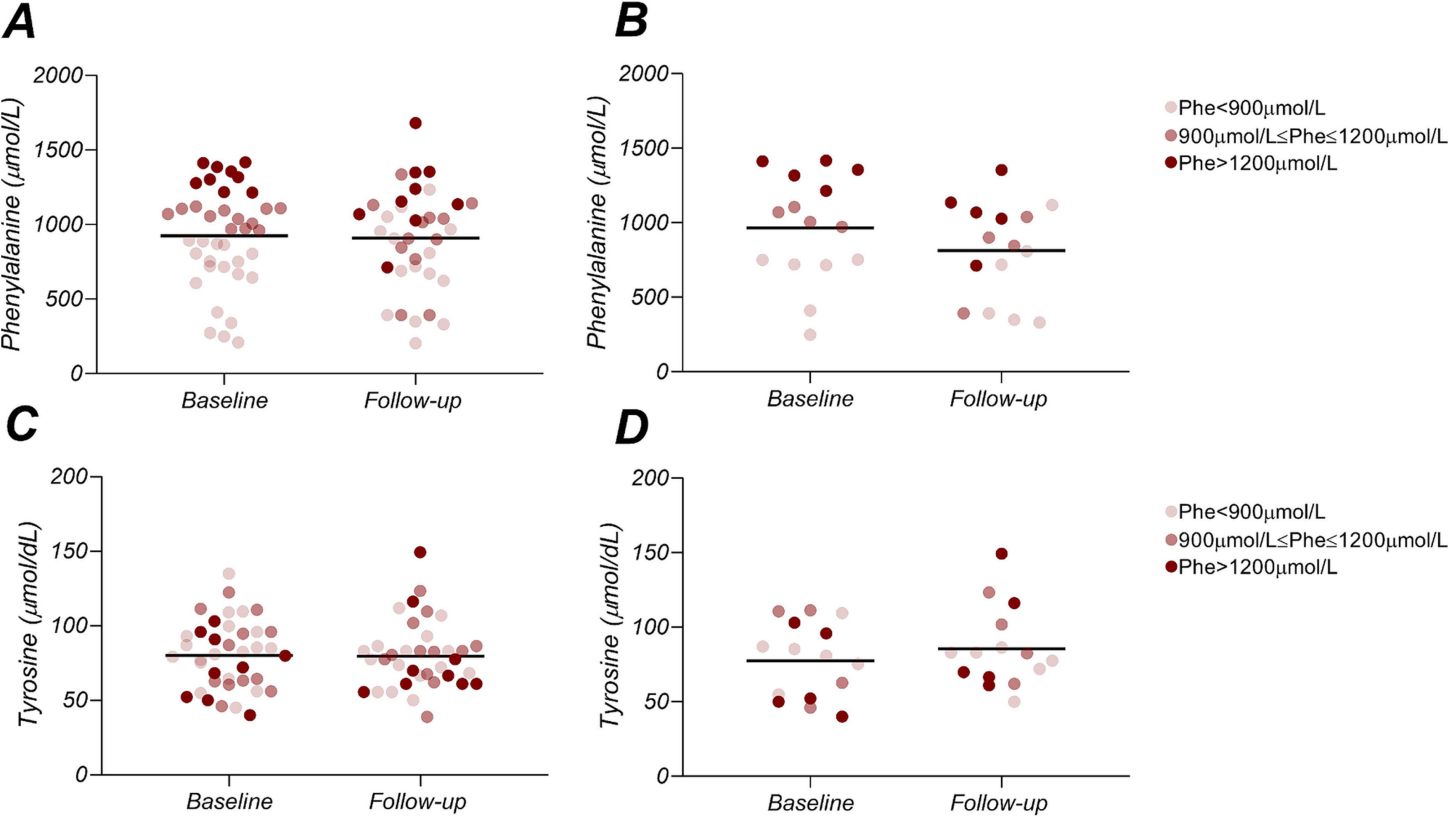
Baseline and follow‐up plasma concentrations of phenylalanine (Phe) and tyrosine (Tyr) in PKU patients participating in the baseline study (*N =* 39) (A and C) and follow‐up study (*N =* 15) (B and D). Phenylalanine and tyrosine values are unchanged at follow‐up; however, these values can vary widely in some patients. Plasma concentrations of lipids in PKU patients in the baseline study (*N* = 39) (E to I) and follow‐up study (*N* = 15) (J to N) of, in order, respectively: total cholesterol, triglycerides, HDL cholesterol, LDLcholesterol, LDL/HDL cholesterol ratio. Categorical classification of PKU patients based on baseline phenylalanine concentrations: • Phe < 900 μmol/L, • 900μmol/L ≤ Phe ≤ 1200 μmol/L, • Phe > 1200 μmol/L. Lipid plasma concentrations are largely unchanged on average at follow‐up.

Phe and Tyr concentrations at baseline and follow‐up did not demonstrate systematic variations (Figure 2 and Table 1): Mean Phe and Tyr concentrations remained unchanged, for the whole cohort of patients included in the initial study, at baseline and follow‐up (Phe 924 ± 330 vs. 909 ± 338 μmol/L, *p* = 0.24, Tyr (80 ± 23 vs. 80 ± 23 μmol/L, *p* = 0.78, *N* = 39) and the subgroup of patients with a CMR evaluation at baseline and follow‐up (Phe 967 ± 360 vs. 815 ± 326 μmol/L, *p* = 0.06, Tyr: 77 ± 25 vs. 85 ± 27 μmol/L, *p* = 0.39, *N* = 15), respectively. The number of patients in each categorical classification of therapeutic efficacy at baseline, according to local clinical standard of care and in agreement with the current European guidelines [23]: good (Phe ≤ 900 μmol/L *N* = 6/15, 40%), moderate (900 μmol/L < Phe ≤ 1200 μmol/L *N* = 4/15, 27%), to be improved (Phe > 1200 μmol/L, *N* = 5/15, 33%), is indicated in Figure 2A, as are the individual changes from the baseline categorial groups, indicating the intrasubject variability over time. Similarly, the lipid concentrations remained unchanged between baseline and follow‐up in both the entire cohort of patients (*N* = 39) and the follow‐up cohort (*N* = 15). (Numerical details presented in Table 1 and illustrated in Figure 2).

**TABLE 1:** Characteristics of study population (baseline and follow‐up).

	PKU patients (*N* = 15)		
	Baseline	Follow‐up	*p*
**Demographics**			
Age, years	31.5 ± 8.1	39.8 ± 8.1	
Males, *n* (%)	9 (60%)	9 (60%)	
**Anthropometrics**			
Height (cm)	173 ± 11	173 ± 11	
Weight, kg	77 ± 14	84 ± 20	***0.029***
BMI, kg/m^2^	25.7 ± 3.8	28.7 ± 6.3	***0.016***
BSA, m^2^	1.92 ± 0.22	2.00 ± 0.26	***0.027***
**Bioelectrical impedance**			
Fat mass, kg	21.6 ± 9.7	21.2 ± 1.5	0.62
Fat‐free mass, kg	57.0 ± 11.7	58.2 ± 13.0	0.45
Ratio	0.39 ± 0.19	0.38 ± 0.19	0.47
Visceral fat, kg	6.1 ± 2.6	7.1 ± 3.7	0.13
BMR, J/(h·kg)	1706 ± 329	1732 ± 372	0.54
Muscle mass	54.2 ± 11.2	55.5 ± 12.4	0.42
Body water	41.7 ± 8.6	42.8 ± 9.6	0.35
**Clinical**			
Heart rate, bpm	66 ± 10	64 ± 12	0.62
Systolic BP, mmHg	115 ± 17	114 ± 19	0.82
Diastolic BP, mmHg	71 ± 10	68 ± 12	0.54
Pulse pressure, mmHg	44 ± 12	46 ± 12	0.75
**Biochemistry**			
Phe mean, μmol/L	967 ± 360	815 ± 326	0.06
Phe median, μmol/L	968 ± 357	818 ± 324	0.06
Phe SD, μmol/L	192 ± 102	204 ± 95	0.48
Tyr mean, μmol/L	77 ± 25	85 ± 27	0.39
Tyr median, μmol/L	77 ± 25	85 ± 27	0.37
Tyr SD, μmol/L	32 ± 16	34 ± 17	0.74
Carnitin free, mg/dL	37.9 ± 11.3	41.6 ± 11.7	0.22
Carnitin total, mg/dL	53.6 ± 9.6	55.0 ± 14.9	0.93
Carnitin ratio free/total	0.71 ± 0.17	0.76 ± 0.12	0.18
Cholesterol total, mg/dL	176 ± 39	163 ± 34	0.10
HDL‐cholesterol, mg/dL	49 ± 13	47 ± 11	0.56
LDL‐cholesterol, mg/dL	102 ± 27	101 ± 26	0.79
Triglycerides, mg/dL	180 ± 120	149 ± 65	0.30
Glucose, mg/dL	74 ± 7	79 ± 12	0.27
	PKU patients ‐ entire cohort (*N* = 39)		
	Baseline	Follow‐up	P Value
Phe mean, μmol/L	924 ± 330	909 ± 338	0.24
Tyr mean, μmol/L	80 ± 23	80 ± 23	0.78
Cholesterol total, mg/dL	162 ± 34	159 ± 34	0.73
HDL‐cholesterol, mg/dL	48 ± 13	48 ± 11	0.75
LDL‐cholesterol, mg/dL	92 ± 25	95 ± 28	0.30
Triglycerides, mg/dL	150 ± 95	148 ± 64	0.72

*Note:* Phe and Tyr are for baseline averaged for a 10‐year period for baseline and for a 2‐year period for follow‐up. Values in bold and italic emphases indicate the *p*‐values.

Abbreviations: BMI, body mass index; BMR, basal metabolic rate; BP, blood pressure; BSA, body surface area; Phe, phenylalanine; Tyr, tyrosine.

Fifteen of thirty‐nine (38.5%) patients participated in the follow‐up CMR with a mean follow‐up period of 8.3 ± 0.3 years. The general characteristics of these patients at baseline and follow‐up are specified in Table 1. At baseline, PKU patients had a slightly higher BMI than control (25.7 ± 5.0 vs. 23.7 ± 3.3, *p* = 0.030, *N* = 39), with 7/39 (18%) being obese (according to the WHO guidelines [24] BMI ≥ 30 kg/m^2^) and none severely obese (BMI ≥ 40 kg/m^2^). Of the fifteen patients of the follow‐up study, 2/15 (13%) were obese at baseline, 4/15 (27%) and 1/15 (7%) were obese and severely obese, respectively, at follow‐up. This was reflected in an increase of BMI (25.7 ± 3.8 vs. 28.7 ± 6.3, *p* = 0.016, *N* = 15, baseline vs. follow‐up, respectively). Bioelectrical impedance analysis showed no significant difference in body distribution of fat, muscle or water but a noticeable trend of an elevated visceral fat component from baseline to follow‐up (*N* = 15).

### 3.2 Cardiac Anatomy, Function and Structural Modifications in PKU Patients

**FIGURE 3:**
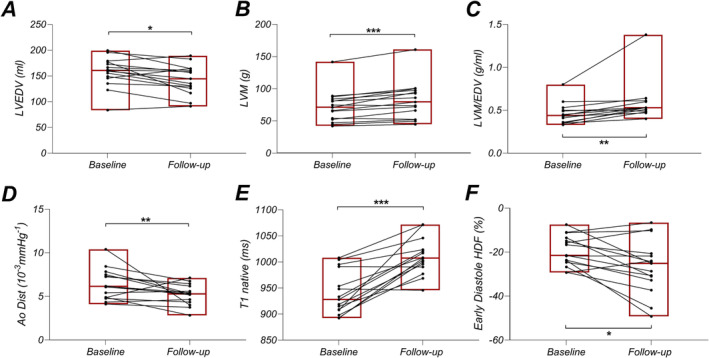
Cardiac structural and functional remodelling in adult patients with phenylketonuria at baseline and follow‐up: (A) LV end‐diastolic volume, (B) LV mass, (C) ratio LV mass to end‐diastolic volume, (D) aortic distensibility, (E) T1 native, (F) early diastolic hemodynamic force. A *p* < 0.05 was considered statistically significant. *p < 0.05, **p < 0.01, ***p < 0.001.

A significant increase in body weight and implicitly BMI and body surface area (BSA) between baseline and follow‐up may introduce a co‐variate in the paired comparisons performed; we present the data under unindexed format (in detail in Table 2). Compared to baseline, at follow‐up, PKU patients (*N* = 15) had lower LV EDV (158 ± 29 vs. 143 ± 29 mL, *p* = 0.013) (Figure 3A) and lower end‐systolic volume (ESV) (68 ± 18 vs. 62 ± 18 mL, *p* = 0.011). There was a nonsignificant trend towards a lower stroke volume (90 ± 17 vs. 81 ± 16 mL, *p* = 0.08) but no change in EF (57 ± 6 vs. 57 ± 7%, *p* = 0.90) which at follow‐up was borderline lower than the expected average value for this age group [25]. LV mass (72 ± 25 vs. 82 ± 29 g, *p* < 0.001 (Figure 3B) and LV septal and lateral wall thickness (septum: 7.0 ± 1.7 vs. 7.2 ± 1.8 mm, *p* = 0.006, lateral wall: 6.1 ± 1.5 vs. 6.2 ± 1.5 mm, *p* = 0.011) were increased, and thus, the LV mass/EDV index was largely increased at follow‐up (0.46 ± 0.12 vs. 0.58 ± 0.23 g/mL, *p* = 0.005) (Figure 3C), indicating a more concentric pattern of the LV remodelling compared to baseline. RV EDV (167 ± 36 vs. 156 ± 36 mL, *p* = 0.049) and RV SV (87 ± 17 vs. 78 ± 17 mL, *p* = 0.022) followed a similar pattern and decreased at follow‐up compared with baseline. In contrast to LV ESV, RV ESV (80 ± 24 vs. 78 ± 22 mL, *p* = 0.23) did not change significantly. Aortic distensibility decreased at follow‐up (6.38 ± 1.75 vs. 5.21 ± 1.17 10^−3^ mmHg^−1^, *p* = 0.008) (Figure 3D). Importantly, T1 times, shorter than control at baseline, increased at follow‐up (940 ± 42 vs. 1010 ± 35 ms, *p* < 0.001) (Figure 3E).

**TABLE 2:** CMR parameters: Volumes, parametric (baseline and follow‐up).

	PKU patients (*N* = 15)		
	Baseline	Follow‐up	*p*
**Left ventricle**			
LV EDV, mL	158 ± 29	143 ± 29	***0.013***
LV ESV, mL	68 ± 18	62 ± 18	***0.011***
LV SV, mL	90 ± 17	81 ± 16	0.08
LV EF, %	57 ± 6	57 ± 7	0.90
LV CO, L/min	6.15 ± 1.21	6.01 ± 1.56	0.68
Septal ED WT, mm	7.0 ± 1.7	7.2 ± 1.8	***0.006***
Lateral ED WT, mm	6.1 ± 1.5	6.2 ± 1.5	***0.011***
Relative WT	1.2 ± 0.1	1.2 ± 0.1	0.72
LV Mass, g	72 ± 25	82 ± 29	***<0.001***
LV Mass/EDV, g/mL	0.46 ± 0.12	0.58 ± 0.23	***0.005***
LV ED maximal diameter, mm	53 ± 5	53 ± 5	0.65
**Left atrium**			
LA Volume max, mL	64 ± 12	67 ± 14	0.42
LA Volume min, mL	20 ± 7	22 ± 7	0.32
LA emptying fraction %	70 ± 6	68 ± 6	0.37
**Right ventricle**			
RV EDV, mL	167 ± 36	156 ± 36	***0.049***
RV ESV, mL	80 ± 24	78 ± 22	0.23
RV SV, mL	87 ± 17	78 ± 17	***0.022***
RV ejection fraction, %	52 ± 6	51 ± 4	0.24
RV CO, mL	5.89 ± 1.03	5.79 ± 1.41	0.70
**Ascending aorta**			
Systolic aortic area, cm^2^	4.53 ± 0.88	4.53 ± 0.92	0.99
Diastolic aortic area, cm^2^	3.58 ± 0.77	3.72 ± 0.87	0.10
Ao distens, 10–3 mmHg^−1^	6.38 ± 1.75	5.21 ± 1.17	***0.008***
**Parametric imaging**			
T1 native, ms	940 ± 42	1010 ± 35	***<0.001***
ECV (%)	26.5 ± 2.6	26.5 ± 3.7	0.77
T2, ms		48.9 ± 3.1	

*Note:* Values in bold and italic emphases indicate the *p*–values. Abbreviations: CO, cardiac output; ECV, extracellular volume; ED, end‐diastolic; EDV, end‐diastolic volume; ES end‐systolic; ESV end‐systolic volume; LA, left atrium; LV, left ventricle; RV, right ventricle; WT wall thickness.

When compared to matched controls (complete data are presented in Table S1), the PKU patients at follow‐up maintained important traits observed at baseline: a lower EF, lower LV mass and thinner LV lateral wall (however, this was not the case of LV septum) and a larger LV end‐diastolic diameter and a reduced RV EF. LA EF in PKU patients remained higher than in Control, in keeping with what we had observed at baseline. Surprisingly, T1 native times were paradoxically even longer than in control, in complete opposition with what was observed at baseline.

### 3.3 Assessment of Cardiac Deformation and Hemodynamic Forces in PKU Patients

To further analyse the changes in cardiac function, we assessed the systolic peak deformation (strain) of the myocardium at multiple layer levels. We also derived the hemodynamic forces for systole, early and late diastole. The detailed numerical values are shown in Table 3. GLS and global circumferential strain (GCS) were not different from baseline at neither subendocardial (Endo) (GLS: −26.5 ± 5.6 vs. −26.9 ± 5.8%, *p* = 0.60, GCS: −30.0 ± 5.3 vs. −31.1 ± 5.2%, *p* = 0.13) nor midmyocardial (Myo) (GLS: −25.3 ± 2.9 vs. −24.9 ± 3.1%, *p* = 0.50, GCS: −20.5 ± 2.8 vs. −20.7 ± 2.5%, *p* = 0.61) levels. In contrast, both GLS and GCS Endo‐subepicardial (Epi) gradients were more pronounced at follow‐up (−3.0 ± 6.0 vs. −4.3 ± 6.0%, *p* = 0.030 and −15.3 ± 5.4 vs. −16.9 ± 5.5, *p* = 0.036)and were not different from the gradients assessed in control (Table S2). However, PKU patients maintained at follow‐up reduced Endo and Myo GCS, compared with control, in keeping with consistently reduced systolic function which persists over time (Table S2).

**TABLE 3:** CMR parameters: Strain, haemodynamic forces (baseline and follow‐up).

	PKU patients (*N* = 15)		
	Baseline	Follow‐up	*p*
**Left ventricle**			
GLS endo, %	‐26.5 ± 5.6	−26.9 ± 5.8	0.60
GLS myo, %	−25.3 ± 2.9	−24.9 ± 3.1	0.50
GLS endo‐epi gradient, %	−3.0 ± 6.0	−4.3 ± 6.0	***0.030***
GCS endo, %	−30.0 ± 5.3	−31.1 ± 5.2	0.13
GCS myo, %	−20.5 ± 2.8	−20.7 ± 2.5	0.61
GCS endo‐epi gradient, %	−15.3 ± 5.4	−16.9 ± 5.5	***0.036***
HD syst force (%)	34.1 ± 9.6	33.4 ± 8.0	0.83
HD syst work (%)	4.3 ± 1.5	5.0 ± 2.4	0.30
HD syst work (mJ)	3.9 ± 1.3	4.1 ± 1.5	0.62
HD syst power (mJ/s)	47.0 ± 22.7	48.8 ± 20.6	0.77
HD early diast force (%)	−19.4 ± 7.0	−26.5 ± 12.2	***0.012***
HD early diast work (%)	−1.3 ± 0.5	−1.4 ± 1.0	0.57
HD early diast work (mJ)	−1.1 ± 0.42	−1.2 ± 0.8	0.69
HD early diast power (mJ/s)	−18.6 ± 7.6	−20.1 ± 13.5	0.59
HD late diast force (%)	−6.8 ± 3.0	−12.1 ± 6.2	***0.004***
**Left atrium**			
LA strain, %	43.8 ± 11.3	37.3 ± 9.6	***0.031***
**Right ventricle**			
RV longitudinal strain, %	−26.7 ± 3.4	−34.1 ± 5.2	***<0.001***

*Note:* Values in bold and italic emphases indicate the *p*–values. Abbreviations: Diast, diastolic; Endo, subendocardial; Epi, subepicardial layers; GCS global circumferential strain; GLS, global longitudinal strain; HD, hemodynamic; LA, left atrium; LV, left ventricle; Myo, midmyocardium; RV, right ventricle; Syst, systolic.

Hemodynamic (HD) early diastolic force had markedly lower negative values (−19.4 ± 7.0 vs−26.5 ± 12.2%, *p* = 0.012) (Figure 3F) at follow‐up. Of note, in the baseline study, HD early diastolic force, measured in all 39 PKU patients was already lower than in matched controls (−24.3 ± 9.8 vs. −17.6 ± 9.0, *p* = 0.002, *N* = 39), indicating higher filling pressures. In addition, at follow‐up, HD late diastolic force had markedly lower negative values (−6.8 ± 3.0 vs. −12.1 ± 6.2%, *p* = 0.004, *N* = 15), LA strain was lower (43.8 ± 11.3 vs. 37.3 ± 9.6%, *p* = 0.031, *N =* 15) and RV longitudinal strain was also more pronouncedly negative (−26.7 ± 3.4 vs. −34.1 ± 5.2, *p* < 0.001, *N =* 15). Moreover, when compared with healthy control, PKU patients had more markedly negative HD early diastolic force, power and work (Table S2). Taken together, these changes suggest an impaired diastolic hemodynamics with increased ventricular and atrial filling pressures in PKU patients that is more severe than a physiological increase in LV diastolic stiffness and increased filling pressures observed with normal aging.

### 3.4 Relation Between Phe Levels and Cardiac Phenotype

**FIGURE 4:**
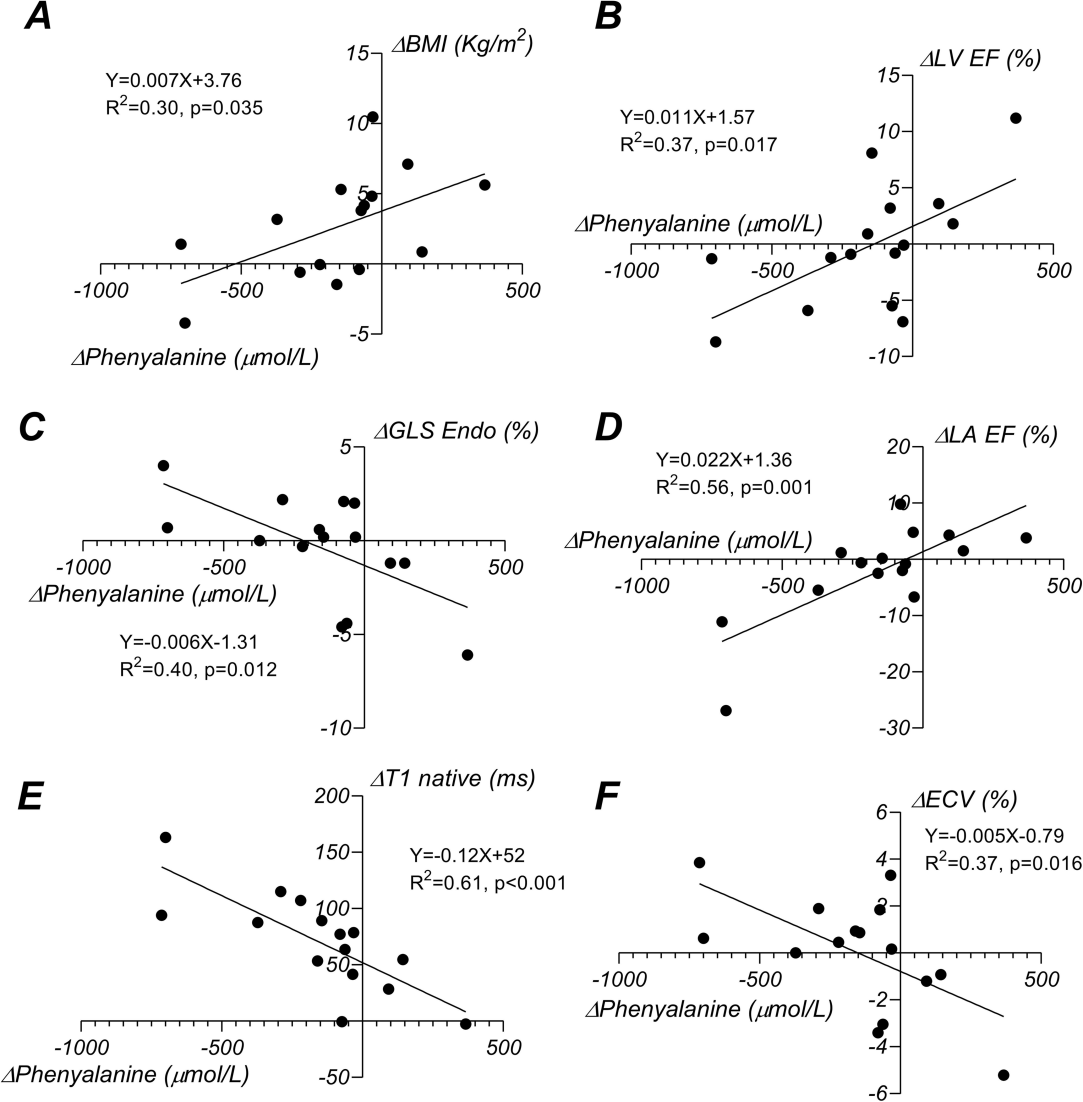
Relation between variation (Δ) between phenylalanine plasma concentrations between baseline (averaged over 10 years) and follow‐up (averaged over 2 years) and variation (Δ) in (A) body mass index (BMI), (B) left ventricular ejection fraction (LV EF), (C) global longitudinal strain measured subendocardially (GLS Endo), (D) left atrial emptying fraction (LA EF), (E) T1 native times, (F) extracellular volume (ECV). Bivariate linear regression indicate that a larger decrease in plasma concentrations of phenylalanine between follow‐up and baseline associate lower ejection fraction, more impaired longitudinal strain, prolonged T1 native times and increased extracellular volume (taken together these likely represent a negative cardiac remodelling with likely more fibrosis and depressed systolic contraction, in patients with a higher decrease in phenylalanine levels. Additionally, higher BMI increase is correlated to a proportional increase in phenylalanine concentrations which may be related to a stricter compliance with food restriction and diet control.

To clarify whether the Phe and Tyr concentrations correlated with cardiovascular changes, we compared the differences between baseline and follow‐up (Δ) for the Phe and Tyr concentrations to those observed in cardiovascular parameters, respectively. These data are presented in Table 4. ΔPhe but not ΔTyr was positively correlated with weight gain (*β* = 0.56, *p* = 0.031) and corollary with BMI (*β* = 0.55, *p* = 0.035) and the BSA (*β* = 0.58, *p* = 0.023) (Figure 4A). Δ Phe correlated moderately with Δ LV EF (*β* = 0.61, *p* = 0.017) (Figure 4B), Δ LV GLS Endo (*β* = −0.63, *p* = 0.012) (Figure 4C) and Δ ECV (*β* = −0.61, *p* = 0.016) (Figure 4F) and strongly with Δ T1 native (*β* = −0.78, *p* < 0.001) (Figure 4E) and with LA emptying fraction (*β* = 0.75, *p* = 0.001) (Figure 4D). Taken together these data suggest that a higher decrease in Phe levels associates with a maladaptive cardiac phenotype at follow‐up indicated by impaired LV systolic function, increased interstitial diffuse collagen deposition (evidence supported by prolonged T1 times and increased ECV) and lower atrial emptying (likely in relation to an increased ventricular stiffness but also directly to atrial fibrosis). The above correlations were maintained with patient age [26, 27] and BMI as cofactors in multivariate regressions (LVEF: *β* = 0.56, p = 0.012, GLS Endo: *β* = −0.63, *p* = 0.011, T1 native: *β* = −0.77, *p* = 0.002, ECV: *β* = −0.61, *p* = 0.014) (Table S3) In contrast with Δ Phe, Δ Tyr did not correlate with any of the parameters evaluated by this study. As the amplitude and mechanisms of cardiac remodelling are distinct between males and females, we tested the interaction between the sex and Δ Phe using a general linear model. Where this interaction was statistically significant, we further perform linear regression in both subgroups of PKU patients: males and females. The associations between Δ Phe and respectively LV EF, GLS Endo and T1 native were stronger in the male patients and not significant tested among the female patients. The complete data are presented in Table S4.

**TABLE 4:** Influence of long‐term variation of phenylalanine and tyrosine on the variation in CMR parameters between baseline and follow‐up intervals.

	Phenylalanine gradient		Tyrosine gradient	
	*β*	*p*	*β*	*p*
**Anthropometrics**				
Δ Weight, kg	0.56	***0.031***	−0.29	0.29
Δ BMI, kg/m^2^	0.55	***0.035***	−0.30	0.28
Δ BSA, m^2^	0.58	***0.023***	−0.30	0.28
**Left ventricle**				
Δ LV EDV, mL	0.11	0.69	0.02	0.94
Δ LV ESV, mL	0.51	***0.050***	0.14	0.62
Δ LV SV, mL	0.34	0.22	−0.04	0.88
Δ LV EF, %	0.61	***0.017***	0.08	0.78
Δ LV CO, L/min	0.28	0.31	−0.16	0.56
Δ Septal ED WT, mm	−0.42	0.12	0.05	0.86
Δ Lateral ED WT, mm	−0.35	0.38	0.22	0.44
Δ LV mass (g)	−0.15	0.59	−0.08	0.76
Δ LV mass/ED volume, g/mL	−0.06	0.83	−0.02	0.94
Δ GLS endo, %	−0.63	***0.012***	0.35	0.20
Δ GCS endo, %	−0.28	0.32	0.39	0.15
Δ HD syst force (%)	−0.17	0.54	0.11	0.70
Δ HD syst work (%)	0.01	0.98	0.04	0.89
Δ HD syst work (mJ)	−0.12	0.66	0.14	0.62
Δ HD syst power (mJ/s)	−0.19	0.50	0.16	0.58
Δ HD early diast force (%)	−0.06	0.82	0.35	0.21
Δ HD early diast work (%)	0.06	0.85	0.19	0.51
Δ HD early diast work (mJ)	0.23	0.40	0.05	0.87
Δ HD early diast power (mJ/s)	0.40	0.14	0.05	0.86
Δ HD late diast force (%)	0.08	0.78	0.04	0.88
**Left atrium**				
Δ LA max Vol, mL	−0.24	0.39	0.41	0.13
Δ LA emptying fraction %	0.75	***0.001***	−0.12	0.67
Δ LA strain, %	0.51	0.05	0.21	0.46
**Right ventricle**				
Δ RV EDV, mL	0.27	0.33	−0.04	0.90
Δ RV ESV, mL	0.10	0.73	−0.41	0.13
Δ RV SV, mL	0.27	0.34	0.15	0.60
Δ RV EF, %	0.21	0.46	0.30	0.27
Δ RV CO, L/min	0.20	0.47	0.07	0.80
Δ RV GLS, %	−0.17	0.55	−0.02	0.96
**Ascending aorta**				
Δ Ao distens, 10‐3 mmHg‐1	−0.11	0.70	0.51	0.05
**Parametric imaging**				
Δ T1 native, ms	−0.78	***<0.001***	−0.02	0.96
Δ ECV, %	−0.61	***0.016***	0.32	0.24
**Biochemistry**				
Δ Carnitin free, mg/dL	0.07	0.82	0.20	0.47
Δ Carnitin total, mg/dL	0.08	0.79	0.19	0.50
Δ Carnitin ratio free/total	0.13	0.64	−0.09	0.75
Δ Cholesterol total, mg/dL	−0.08	0.78	−0.22	0.42
Δ HDL‐cholesterol, mg/dL	−0.02	0.94	0.06	0.84
Δ LDL‐cholesterol, mg/dL	0.00	0.99	0.41	0.13
Δ Triglycerides, mg/dL	−0.13	0.65	−0.40	0.14

*Note:* As ΔPhe is correlated to estimated ΔBSA, measured and not indexed values of CMR parameters were included in this analysis. Values in bold and italic emphases indicate the *p*‐values.

Abbreviations: Ao, disten aortic distensibility; BMI, body mass index; BSA, body surface area; CO, cardiac output; Diast, diastolic; ECV, extracellular volume; EDV, end‐diastolic volume; Endo, subendocardial; ESV end‐systolic volume; GCS, global circumferential strain; GLS, global longitudinal strain; HD, hemodynamic; LA, left atrium; LV, left ventricle; RV right ventricle; SV, stroke volume; Syst, systolic; WT wall thickness.

## 4 Discussion

In this study, we present new findings on cardiac remodelling in adult patients with PKU from a longitudinal investigation spanning a mean of 8.3 ± 0.3 years. Upon follow‐up, there was, on average, an increase in BMI but no significant change in body composition ratios was noted. Cardiac remodelling observed initially, characterised by a dilated LV cavity and markedly reduced LV mass compared to healthy individuals, persisted at follow‐up, albeit shifting towards a more concentric pattern. LV EF and GCS showed no significant change from baseline but remained lower than in the control group. Diastolic HD forces were notably more negative in PKU patients, consistent with trends observed at baseline. Interestingly, a more pronounced maladaptive cardiac remodelling, evidenced by lower EF and less markedly reduced GLS, and higher T1 native times and ECV, correlated with a more significant decrease in Phe levels from baseline to follow‐up. Adult patients with PKU undergo regular monitoring of their Phe concentrations and are advised to maintain its values within the therapeutic ranges outlined by national guidelines or local protocols [28]. For the cohort of patients analysed here, the recommended target range was 1200 μmol/L. It was observed previously that, despite medical recommendations, support and personal effort, in some individuals, these values can fluctuate over years with the underlying clinical reasons and implications not fully understood. Some studies suggest that in addition to maintaining low plasma concentrations, the stability of Phe levels over time may play a crucial role in preventing neurological deterioration and functional complications associated with PKU [29]. However, the pathophysiological mechanisms explaining how variable Phe concentrations contribute to tissue damage are still unclear. In this investigation, although there was a notable decline in the mean Phe concentration during follow‐up, this change did not reach statistical significance due to considerable variability among individuals. In contrast, tyrosine (Tyr) concentrations remained more stable, likely due to ongoing supplementation therapy guided by periodic plasma level monitoring.

To obtain a more reliable representation of long‐term, stable Phe concentrations, averaged values over a 2‐year period of Phe, were used for both baseline and follow‐up calculations. This approach should have (i) corrected for the short‐term fluctuations in plasma concentrations which are less important from a clinical perspective and are primarily caused by arbitrary factors such as timing of the blood sample collection, recent food intake composition or bodily activities, and (ii) provided insight into the long‐term variability of these concentrations, which likely reflects changes in adherence to a restrictive diet or other chronic biochemical modifications associated with the disease (prolonged oxidative stress, dyslipidemia, interferences with cellular metabolism, etc.). Despite these measures, wide intraindividual variations were still observed. While the statistical issues related to interindividual variations with only 15 patients might be mitigated by a higher number of patients, a more homogeneous Phe concentration could have solved the statistical problem. However, this would likely mask the effects of these variations and their cardiovascular manifestation.

As part of the current clinical guidelines, PKU patients are advised to control long‐term Phe intake as much as possible by restricting food rich in proteins such as meat, milk, dairy and eggs [2]. Even if national and local practices regarding the acceptable range vary, achieving lower Phe concentrations through adherence to a Phe‐free diet is widely considered a positive therapeutic goal [30]. However, despite controlled nutritional adjustments under specialist guidance, a protein‐depleted food intake profile tends towards a lipid and carbohydrate‐dominated high‐caloric diet, resulting in higher BMI and particularly higher fat/muscular tissue mass [31]. Lipid abnormalities are frequently seen in adult patients with PKU [32] where a diet rich in unsaturated fats and carbohydrates to compensate for a relatively low protein content can lead to abnormal lipid metabolism. Prolonged dyslipidemia has been linked to cardiac systolic and diastolic contractile deficit induced through direct effects on SERCA‐2 expression, intracellular Ca^2+^ handling, and structural modifications such as cardiac lipidosis [33]. In our cohort, we found higher triglycerides, lower HDL cholesterol, and consequently, a much higher LDL/HDL cholesterol ratio both at baseline and follow‐up compared to the healthy matched controls. These changes in lipid metabolism could be causally related to the observed cardiac tissue changes.

At baseline, we found that PKU patients had a more dilated LV with thinner walls and a severely reduced myocardial mass compared with matched healthy controls. These differences were maintained at follow‐up. However, we noted a relative decrease in EDV and a modest, yet notable, relative increase in LV mass. Consequently, the ratio LVM/EDV—an index used to discriminate between concentric and dilatative remodelling patterns—was markedly increased between baseline and follow‐up, reaching levels similar to those observed in the control group, despite the maintained lower LV mass in PKU patients. PKU patients demonstrated very short, abnormal T1 times at baseline. At follow‐up, these values not only increased compared to baseline but were also elevated compared to those measured in control subjects. Taken together with a comparatively high increase in LVM and LVM/EDV ratio, these changes may represent excessive diffuse myocardial fibrosis [34] or a change in the lipid profile such as a lower myocardial triglyceride content [35]. Consistent with this pattern of remodelling, it is likely that a degree of compensatory maladaptive hypertrophy is present in PKU patients in the context of prolonged diastolic and systolic deficits, even if these parameters do not fall outside an expected normal range.

HD diastolic forces represent a promising new imaging marker of increased LV filling pressure and diastolic impairment (Supporting Information S5). In the initial study, examining the PKU cohort at baseline, we found that early diastolic HD force was more markedly negative in PKU patients compared with healthy subjects, indicating early elevated LV filling pressures and diastolic dysfunction [10]. Here, we demonstrate a further decrease at follow‐up compared with baseline levels, suggesting the sustained nature of these changes. Additionally, HD early diastolic force, work and power were more markedly negative in PKU compared to control subjects. LV late diastolic HD force was also more negative, and LA strain was reduced in PKU patients at follow‐up compared to baseline, indicating both higher late ventricular diastolic filling pressure and reduced atrial booster pump function. All these findings support the idea of a progressive worsening of the diastolic function in adult PKU patients, with more strained diastolic hemodynamics. Both mechanical stress, resulting from a prolonged suboptimal contractile performance and elevated reactive oxygen species signalling, could be responsible for increased activation of myocardial fibroblast and excessive diffuse collagen deposition. Aortic distensibility decreased at follow‐up, consistent with recent findings correlating oxidative stress levels with endothelial dysfunction, estimated as vascular impaired flow‐mediated dilatation [13]. In turn, increased arterial stiffness is prone to precipitate and contribute to the development and exacerbation of hemodynamic stress and increasing energy demands on the heart muscle.

EF and both GLS and GCS remained unchanged on average between baseline and follow‐up, suggesting that in many patients, the systolic function is reasonably preserved at this stage, even if an excessive degree of hypertrophic remodelling can mask relatively lower levels of EF. Compared with control subjects, systolic function remains lower in PKU patients, with lower EF and less markedly reduced GCS. Closer examination of paired data indicated that in some patients, these parameters declined abruptly, prompting us to investigate if a relation exists between variations in parameters characterizing cardiac remodelling and variations in Phe and Tyr. Our data indicated a correlation between the decline of Phe concentrations and a lower increase in BMI, likely achieved through better control of body weight via diet. Surprisingly, this was associated with adverse cardiac remodelling, including an increase in T1 times and ECV—both markers of diffuse myocardial fibrosis—and a decrease in LV EF and less markedly reduced GLS, which are indicators of LV systolic function. Moreover, a decrease in LA global emptying fraction, a strong indicator of both atrial remodelling and fibrosis as well as chronically elevated LV filling pressures and diastolic dysfunction, paralleled the increase in T1 and ECV. This strengthens the hypothesis of more pronounced cardiac remodelling in PKU patients with a greater drop in Phe concentrations. In contrast, variations in Tyr did not correspond to any change in cardiac phenotype. In support of the outlined factors of adverse cardiac remodelling, previous studies have demonstrated the importance of a protein‐rich, carbohydrate and lipid‐depleted diet in preventing coronary artery disease and HF [36, 37] (Supporting Information S6). Obese non‐PKU patients who lose weight through a strict control of food intake have a limited improvement in cardiac structure and function compared with subjects who achieved a similar decrease in body mass following bariatric surgery [38]. This points towards the idea that a substrate‐selective diet, particularly protein restriction, could carry unwanted negative effects on cardiovascular physiology [39].

We and others demonstrated a high incidence of proteinuria, suggesting chronic kidney disease, among adult PKU patients, which has been related to excessive dietary amino‐acid supplementations as part of the dietary therapy [40] (Supporting Information S7). In these patients, a sustained decrease in Phe concentrations may be related to an abnormal glomerular filtration and incipient development of nephrotic syndrome, which in turn may be associated with deleterious effects on the cardiovascular system. This intriguing hypothesis will need further testing as a detailed assessment of renal impairment and its effect on cardiovascular phenotype was beyond the scope of our current study. An alternative explanation for excessive protein excretion may be related to prolonged diet amino acid supplementations.

## 5 Limitations

We acknowledge the low number of participants in our follow‐up study which may hide some of the significant differences at follow‐up, larger studies comprising patients with a variety of Phe levels are required to give a more definitive answer. We could not include in our follow‐up study the same cohort of control subjects used in the baseline study; however, we did include a group of matched volunteers selected from our local CMR database. Due to local hardware limitations and ethical constraints, we could not include a comprehensive cardiac spectroscopy examination in our follow‐up visit, and no histology or biomarker data were available to support the structural changes reported using parametric mapping. This could have offered an important insight into the lipid infiltration of the heart, energetic balance and substrate utilisation in the PKU heart and represents an important line of further investigations. Similarly, we could not include an echocardiography examination in the follow‐up visit, and this could have provided a more well‐validated and detailed evaluation of the diastolic function. However, to characterise LV diastolic filling pressure, we used previously validated novel CMR parameters such as diastolic hemodynamic forces.

## 6 Conclusions

Adult PKU patients may carry an increased risk for developing HF compared with the same age group of healthy individuals with persistently lower systolic performance and increased diastolic filling pressure. We show here that a drastic decrease in Phe can be associated with increasingly maladaptive cardiac remodelling and a more marked functional impairment. These new data need further warrant detailed scrutiny of the cardiovascular phenotype in larger cohorts of patients and mechanistic approaches on available disease models.

## Conflicts of Interest

The authors declare no conflicts of interest.

## Supporting information


**Figure S1.** Plasma concentrations of lipids in PKU patients, included all PKU patients participating in the baseline study (*N* = 39) (A to E) of, in order: total cholesterol, triglycerides, HDL cholesterol, LDLcholesterol, LDL/HDL cholesterol ratio. Categorical classification of PKU patients based on baseline phenylalanine concentrations: •Phe < 900 μmol/L, •900 μmol/L ≤ Phe ≤ 12ooμmol/L, • Phe > 1200 μmol/L. Lipid plasma concentrations are unchanged on average at follow‐up.
**Figure S2.** Plasma concentrations of lipids in PKU patients ‐ inlcuded only PKU patients participating in the follow‐up study (*N* = 15) (A to E) of, in order: total cholesterol, triglycerides, HDL cholesterol, LDLcholesterol, LDL/HDL cholesterol ratio. Categorical classification of PKU patients based on baseline phenylalanine concentrations: •Phe < 900 μmol/L, •900 μmol/L ≤ 5Phe ≤ 51200μmol/L, • Phe > 1200 μmol/L. Lipid plasma concentrations are unchanged on average at follow‐up.
**Figure S3.** Supporting Information.

**Table S1.** CMR Parameters: Volumes, Parametric Imging – in PKU patients at follow‐up and a matched Control group.
**Table** **S** **2.** CMR parameters: strain, haemodynamic forces in PKU patients at follow‐up and a matched Control group.
Table S3. Influence of phenylalanine variation on cardiovascular phenotype in PKU patients – univariate (left columns) and co‐variate with BMI and age (right columns).
**Table S4.** Influence of phenylalanine variation on cardiovascular phenotype in PKU patients – significance of interaction between patient sex and phenylalanine variation and cardiac parameters (left), univariate linear regression in the two subgroups of follow‐up PKU patients, males and females (right).
