# Supplementary material for: Large Variations in Phenylalanine Concentrations Associate Adverse Cardiac Remodelling in Adult Patients With Phenylketonuria—A Long‐Term CMR Study
Source: J Cachexia Sarcopenia Muscle. 2025 Jan 10;16(1):e13667. doi: 10.1002/jcsm.13667 (PMC11724155; doi:10.1002/jcsm.13667)
Supplement: Supplementary file 1 — Figure S1. Plasma concentrations of lipids in PKU patients, included all PKU patients participating in the baseline study (N = 39) (A to E) of, in order: total cholesterol, triglycerides, HDL cholesterol, LDLcholesterol, LDL/HDL cholesterol ratio. Categorical classification of PKU patients based on baseline phenylalanine concentrations: •Phe < 900 μmol/L, •900 μmol/L ≤ Phe ≤ 12ooμmol/L, • Phe > 1200 μmol/L. Lipid plasma concentrations are unchanged on average at follow‐up. Figure S2. Plasma concentrations of lipids in PKU patients ‐ inlcuded only PKU patients participating in the follow‐up study (N = 15) (A to E) of, in order: total cholesterol, triglycerides, HDL cholesterol, LDLcholesterol, LDL/HDL cholesterol ratio. Categorical classification of PKU patients based on baseline phenylalanine concentrations: •Phe < 900 μmol/L, •900 μmol/L ≤ 5Phe ≤ 51200μmol/L, • Phe > 1200 μmol/L. Lipid plasma concentrations are unchanged on average at follow‐up. Figure S3. Supporting Information. [file JCSM-16-e13667-s001.pdf]

Supplemental Figure 1.

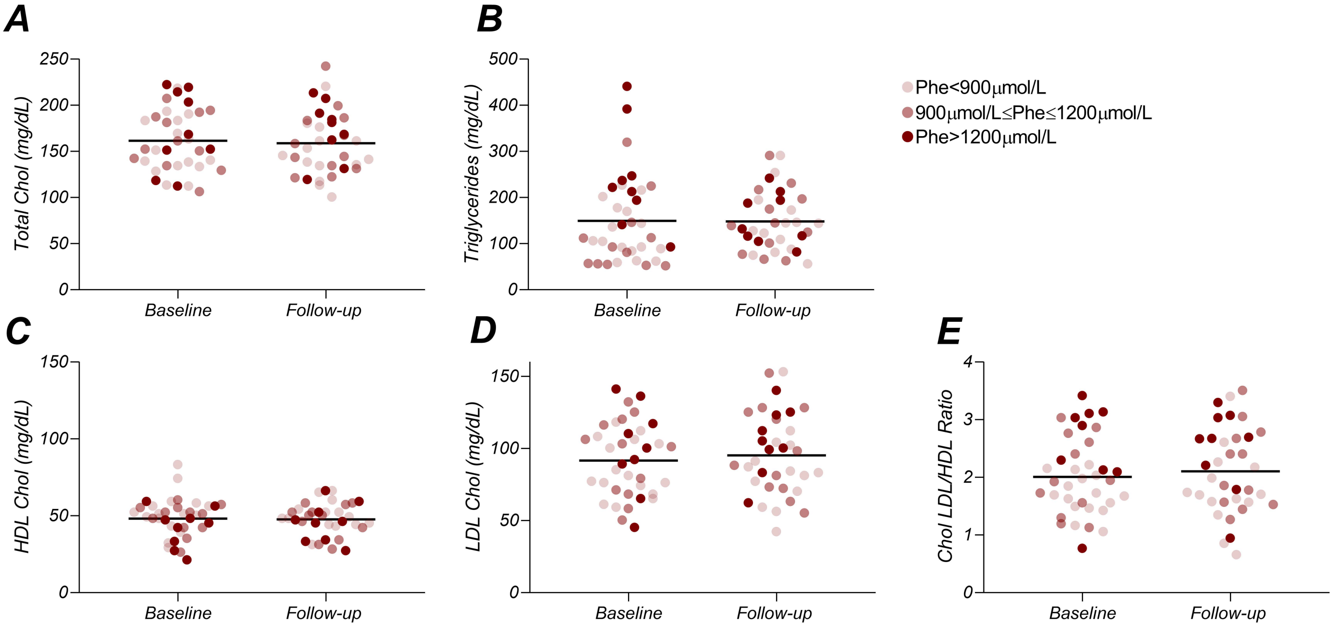

Supplemental Figure 1. Plasma concentrations of lipids in PKU patients, included all PKU patients participating in the baseline study (N=39) (**A** to **E**) of, in order: total cholesterol, triglycerides, HDL cholesterol, LDLcholesterol, LDL/HDL cholesterol ratio. Categorical classification of PKU patients based on baseline phenylalanine concentrations: ●Phe<900μmol/L, ●900μmol/L≤Phe≤1200μmol/L, ● Phe>1200μmol/L. Lipid plasma concentrations are unchanged on average at follow-up.

Supplemental Figure 2.

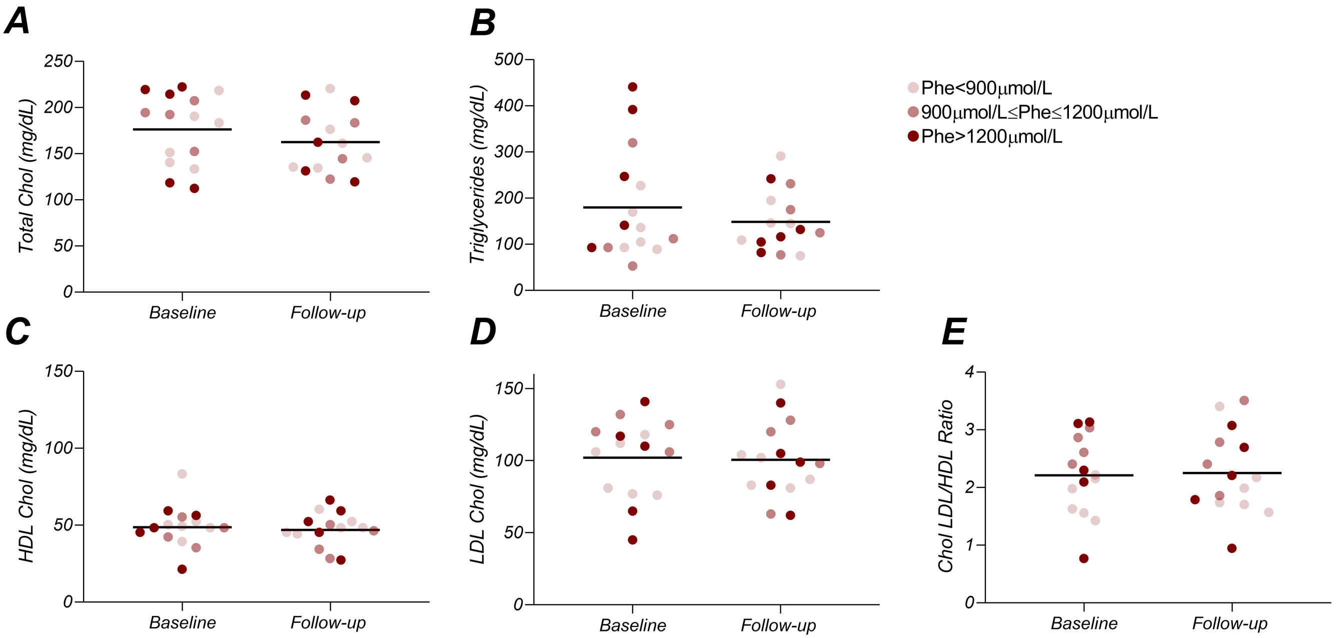

Supplemental Figure 2. Plasma concentrations of lipids in PKU patients - inlcuded only PKU patients participating in the follow-up study (N=15) (**A** to **E**) of, in order: total cholesterol, triglycerides, HDL cholesterol, LDLcholesterol, LDL/HDL cholesterol ratio. Categorical classification of PKU patients based on baseline phenylalanine concentrations: ●Phe<900µmol/L, ●900µmol/L≤Phe≤1200µmol/L, ● Phe>1200µmol/L. Lipid plasma concentrations are unchanged on average at follow-up.

Supplemental Figure 3.

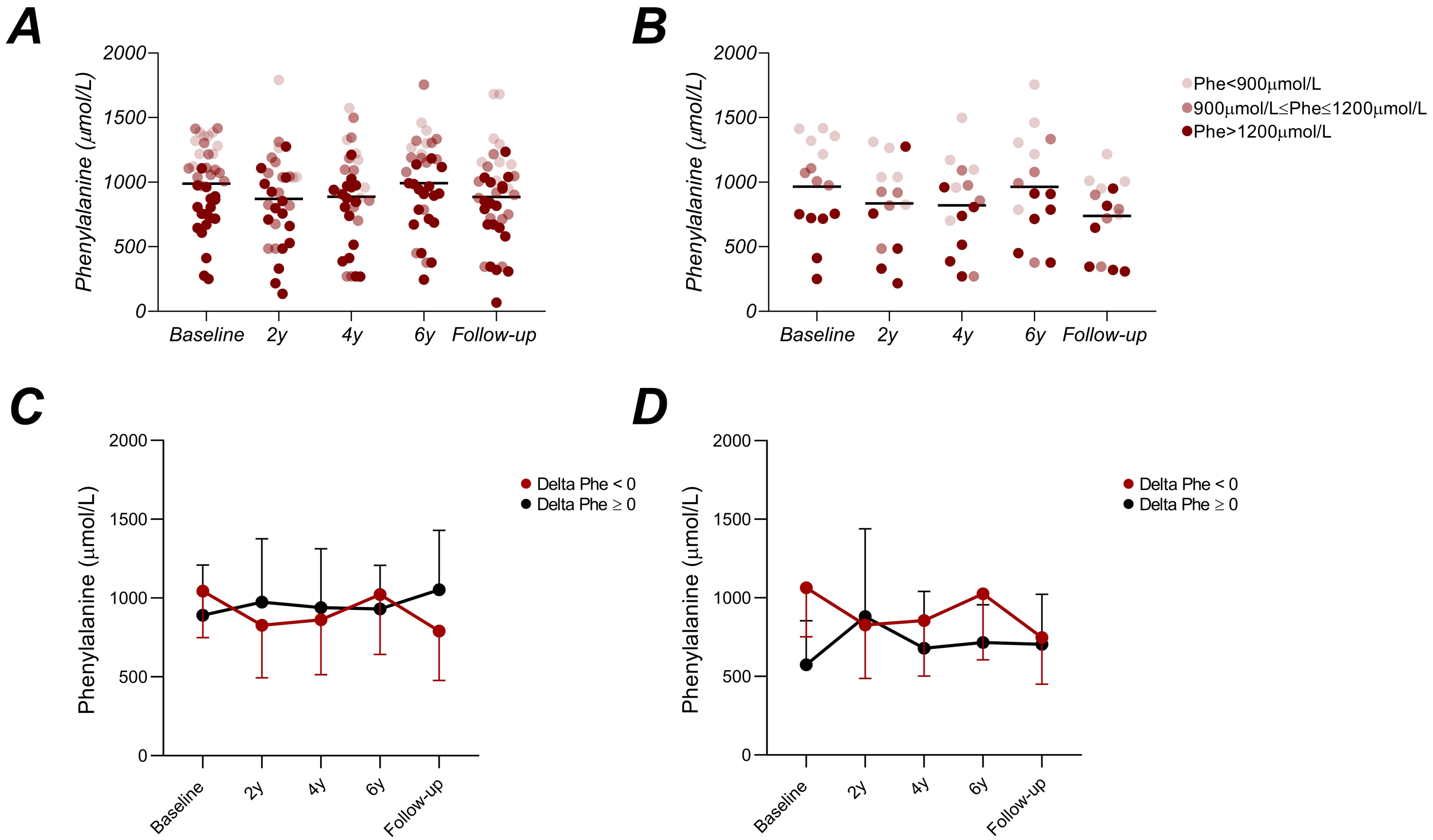

Time Point	Baseline	2 years	4 years	6 years	Follow-up
**All Patients (N=39)**					
Phe Mean±SD	989±308	870±354	888±353	993±349	885±356
Phe - subgroup Δ Phe ≥ 0	891±318	974±402	939±374	930±277	1053±376
Phe - subgroup Δ Phe < 0	1043±294	828±333	862±348	1023±381	791±313
**Follow-up Patients (N=15)**					
Phe Mean±SD	967±360	836±350	820±349	964±404	739±290
Phe - subgroup Δ Phe ≥ 0	574±281	881±559	679±362	716±239	704±318
Phe - subgroup Δ Phe < 0	1065±312	828±340	856±353	1026±420	748±297

Supplemental Table 1. CMR Parameters: Volumes, Parametric Imging – in PKU patients at follow-up and a matched Control group

	PKU patients (N=15)	Control (N=20)	P Value
Age, y	39.8±8.1	40.3±6.6	0.84
Males, n (%)	9(60)	11(55)	0.77
Height, cm	173±11	172±10	0.78
Weight, kg	84±20	75±15	0.14
BMI, kg/m²	28.7±6.3	25.4±3.1	***0.049***
BSA, m²	2.00±0.26	1.89±0.22	0.18
**Left Ventricle**			
LV EDVi, mL/m²	72±13	78±12	0.17
LV ESVi, mL/m²	31±9	29±7	0.46
LV SVi, mL/m²	41±7	47±6	***0.010***
LV EF, %	57±7	62±5	***0.019***
LV CI, L/min/mL/m²	3.0±0.5	2.8±0.5	0.25
Septal ED WT, mm	7.2±1.8	8.1±1.6	0.13
Lateral ED WT, mm	6.2±1.5	7.7±1.1	***0.002***
Relative WT	1.2±0.1	1.1±0.2	0.09
LVMi, g/m²	40±10	50±10	***0.019***
LVM/EDV, g/mL	0.58±0.23	0.54±0.15	0.54
LV ED maximal diameter, mm	53±5	44±4	***<0.001***
**Left Atrium**			
LAVmax index, mL/m²	38±7	40±7	0.41
LAVmin index, mL/m²	14±5	15±3	0.47
LA emptying fraction %	68±6	63±4	***0.006***
**Right Ventricle**			
RV EDVi, mL/m²	78±17	75±17	0.61
RV ESVi, mL/m²	39±11	32±9	***0.046***
RV SVi, mL/m²	40±8	43±10	0.35
RV ejection fraction, %	51±4	58±5	***<0.001***
RV CI, mL/m²	2.9±0.5	2.6±0.7	0.17
**Ascending Aorta**			
Systolic aortic area, cm2	4.53±0.92	4.47±0.85	0.84
Diastolic aortic area, cm2	3.72±0.87	3.70±0.79	0.94
Ao distens, 10-3mmHg-1	5.21±1.17	5.26±1.03	0.89
**Parametric Imaging**			
T1 native, ms	1010±35	972±29	***0.001***
ECV (%)	26.5±3.7	25.1±4.2	0.31
T2, ms	48.9±3.1	48.1±2.0	0.36

Abbreviations: LV left ventricle, EDVi indexed end-diastolic volume, ESVi indexed end-systolic volume, ED end-diastolic, ES end-systolic, CI cardiac index, WT wall thickness, LVM left ventricular mass, LA left atrium, RV right ventricle, ECV extracellular volume.

Supplemental Table 2. CMR Parameters: Strain, Haemodynamic Forces in PKU patients at follow-up and a matched Control group

	PKU patients (N=15)	Control (N=20)	P Value
**Left Ventricle**			
GLS Endo, %	-26.9±5.8	-25.9±3.1	0.52
GLS Myo, %	-24.9±3.1	-24.1±2.3	0.39
GLS Endo-Epi Gradient, %	-4.3±6.0	-4.2±2.4	0.95
GCS Endo, %	-31.1±5.2	-35.7±5.7	***0.020***
GCS Myo, %	-20.7±2.5	-23.8±3.4	***0.005***
GCS Endo-Epi Gradient, %	-16.9±5.5	-19.7±5.0	0.13
HD Syst Force (%)	33.4±8.0	35.0±11.4	0.65
HD Syst Work (%)	5.0±2.4	6.6±3.5	0.14
HD Syst Work (mJ)	4.1±1.5	5.0±2.1	0.17
HD Syst Power (mJ/s)	48.8±20.6	45.4±19.7	0.62
HD Early Diast Force (%)	-26.5±12.2	-11.2±5.5	***<0.001***
HD Early Diast Work (%)	-1.4±1.0	-0.9±0.4	***0.049***
HD Early Diast Work (mJ)	-1.2±0.8	-0.6±0.3	***0.004***
HD Early Diast Power (mJ/s)	-20.1±13.5	-8.2±4.2	***<0.001***
HD Late Diast Force (%)	-12.1±6.2	-9.0±4.5	0.10
**Left Atrium**			
LA strain, %	37.3±9.6	33.1±7.4	0.15
**Right Ventricle**			
RV longitudinal strain, %	-34.1±5.2	-32.3±5.4	0.33

Abbreviations: GLS global longitudinal strain, GCS global circumferential strain, Endo subendocardial, Myo mid-myocardium, Epi subepicardial layers, HD hemodynamic, Syst systolic, Diast diastolic, LV left ventricle, LA left atrium, RV right ventricle

Supplemental Table 3. Influence of phenylalanine variation on cardiovascular phenotype in PKU patients – univariate (left columns) and co-variate with BMI and age (right columns).

	Phenylalanine Gradient		Phenylalanine Gradient controlled for age and BMI	
	β	P Value	β<br>* controlled for age only	P Value
**Anthropometrics**				
Δ Weight, kg	0.56	***0.031***	0.53*	***0.045***
Δ BMI, kg/m2	0.55	***0.035***	0.53*	***0.049***
Δ BSA, m2	0.58	***0.023***	0.56*	***0.034***
**Left Ventricle**				
Δ LV EDV, mL	0.11	0.69		
Δ LV ESV, mL	0.51	***0.050***	0.53	***0.044***
Δ LV SV, mL	0.34	0.22		
Δ LV EF, %	0.61	***0.017***	0.56	***0.012***
Δ LV CO, L/min	0.28	0.31		
Δ Septal ED WT, mm	-0.42	0.12		
Δ Lateral ED WT, mm	-0.35	0.38		
Δ LV Mass (g)	-0.15	0.59		
Δ LV Mass/ED volume, g/mL	-0.06	0.83		
Δ GLS Endo, %	-0.63	***0.012***	-0.63	***0.011***
Δ GCS Endo, %	-0.28	0.32		
Δ HD Syst Force (%)	-0.17	0.54		
Δ HD Syst Work (%)	0.01	0.98		
Δ HD Syst Work (mJ)	-0.12	0.66		
Δ HD Syst Power (mJ/s)	-0.19	0.50		
Δ HD Early Diast Force (%)	-0.06	0.82		
Δ HD Early Diast Work (%)	0.06	0.85		
Δ HD Early Diast Work (mJ)	0.23	0.40		
Δ HD Early Diast Power (mJ/s)	0.4	0.14		
Δ HD Late Diast Force (%)	0.08	0.78		
**Left Atrium**				
Δ LA max Vol, mL	-0.24	0.39		
Δ LA emptying fraction %	0.75	***0.001***	0.77	***0.003***
Δ LA strain, %	0.51	0.05		
Right Ventricle				
Δ RV EDV, mL	0.27	0.33		
Δ RV ESV, mL	0.1	0.73		
Δ RV SV, mL	0.27	0.34		
Δ RV EF, %	0.21	0.46		
Δ RV CO , L/min	0.2	0.47		
Δ RV GLS, %	-0.17	0.55		
**Ascending Aorta**				
Δ Ao distens, 10-3mmHg-1	-0.11	0.70		
**Parametric Imaging**				
Δ T1 native, ms	-0.78	***<0.001***	-0.77	***0.002***
Δ ECV, %	-0.61	***0.016***	-0.61	***0.014***

Abbreviations:BMI body mass index, BSA body surface area, LV left ventricle, EDV end-diastolic volume, ESV end-systolic volume, SV stroke volume, CO cardiac output, WT wall thickness,, GLS global longitudinal strain, GCS global circumferential strain, Endo subendocardial, HD hemodynamic, Syst systolic, Diast diastolic, LA left atrium, RV right ventricle, Ao disten aortic distensibility, ECV extracellular volume. As ΔPhe is correlated to estimated ΔBSA, measured and not indexed values of CMR parameters were included in this analysis. As BMI is correlated with Weight and BSA, the variations in Wight, BMI and BSA were covariate with age only (*).

Supplemental Table 4. Influence of phenylalanine variation on cardiovascular phenotype in PKU patients – significance of interaction between patient sex and phenylalanine variation and cardiac parameters (left), univariate linear regression in the two subgroups of follow-up PKU patients, males and females (right).

	GLM - Interaction	Linear Regression vs Phenylalanine Gradient			
	M-F vs Parameter	Males		Females	
	P Value	β	P Value	β	P Value
**Anthropometrics**					
Δ Weight, kg	***0.036***	0.68	***0.044***	0.19	0.72
Δ BMI, kg/m2	***0.049***	0.65	0.06	0.35	0.50
Δ BSA, m2	***0.015***	0.71	***0.024***	0.12	0.82
**Left Ventricle**					
Δ LV EDV, mL	0.25				
Δ LV ESV, mL	0.07				
Δ LV SV, mL	0.20				
Δ LV EF, %	***0.050***	0.83	***0.005***	-0.05	0.92
Δ LV CO, L/min	0.38				
Δ Septal ED WT, mm	***0.023***	-0.73	***0.024***	0.04	0.94
Δ Lateral ED WT, mm	0.07				
Δ LV Mass (g)	0.42				
Δ LV Mass/ED volume, g/mL	0.89				
Δ GLS Endo, %	***0.049***	-0.75	***0.020***	-0.54	0.27
Δ GCS Endo, %	0.42				
Δ HD Syst Force (%)	0.78				
Δ HD Syst Work (%)	0.99				
Δ HD Syst Work (mJ)	0.81				
Δ HD Syst Power (mJ/s)	0.73				
Δ HD Early Diast Force (%)	0.67				
Δ HD Early Diast Work (%)	0.69				
Δ HD Early Diast Work (mJ)	0.14				
Δ HD Early Diast Power (mJ/s)	0.09				
Δ HD Late Diast Force (%)	0.50				
**Left Atrium**					
Δ LA max Vol, mL	0.34				
Δ LA emptying fraction %	***0.004***	0.79	***0.011***	0.81	***0.049***
Δ LA strain, %	***0.036***	0.25	0.52	0.82	***0.047***
**Right Ventricle**					
Δ RV EDV, mL	0.41				
Δ RV ESV, mL	0.94				
Δ RV SV, mL	0.33				
Δ RV EF, %	0.51				
Δ RV CO , L/min	0.50				
Δ RV GLS, %	0.11				
**Ascending Aorta**					
Δ Ao distens, 10-3mmHg-1	0.84				
**Parametric Imaging**					
Δ T1 native, ms	***<0.001***	-0.94	***<0.001***	-0.63	0.18
Δ ECV, %	0.06				
Δ T2, ms	0.55				

Abbreviations:GLM generalized linear model, BMI body mass index, BSA body surface area, LV left ventricle, EDV end-diastolic volume, ESV end-systolic volume, SV stroke volume, CO cardiac output, WT wall thickness,, GLS global longitudinal strain, GCS global circumferential strain, Endo subendocardial, HD hemodynamic, Syst systolic, Diast diastolic, LA left atrium, RV right ventricle, Ao disten aortic distensibility, ECV extracellular volume. As ΔPhe is correlated to estimated ΔBSA, measured and not indexed values of CMR parameters were included in this analysis

Supplementary References

S1. Macleod EL, Ney DM. Nutritional Management of Phenylketonuria. *Ann Nestle Eng*. 2010;68:58-69.

S2. Reddy YNV, Carter RE, Obokata M, Redfield MM, Borlaug BA. A Simple, Evidence-Based Approach to Help Guide Diagnosis of Heart Failure With Preserved Ejection Fraction. *Circulation*. 2018;138:861-870.

S3. Delles C, Rankin NJ, Boachie C, McConnachie A, Ford I, Kangas A, Soininen P, Trompet S, Mooijaart SP, Jukema JW, et al. Nuclear magnetic resonance-based metabolomics identifies phenylalanine as a novel predictor of incident heart failure hospitalisation: results from PROSPER and FINRISK 1997. *Eur J Heart Fail*. 2018;20:663-673.

S4. Dobrowolski SF, Phua YL, Vockley J, Goetzman E, Blair HC. Phenylketonuria oxidative stress and energy dysregulation: Emerging pathophysiological elements provide interventional opportunity. *Mol Genet Metab*. 2022;136:111-117.

S5. Vallelonga F, Airale L, Tonti G, Argulian E, Milan A, Narula J, Pedrizzetti G. Introduction to Hemodynamic Forces Analysis: Moving Into the New Frontier of Cardiac Deformation Analysis. *J Am Heart Assoc*. 2021;10:e023417.

S6. Voors AA, Anker SD, Cleland JG, Dickstein K, Filippatos G, van der Harst P, Hillege HL, Lang CC, Ter Maaten JM, Ng L, et al. A systems BIOlogy Study to TAilored Treatment in Chronic Heart Failure: rationale, design, and baseline characteristics of BIOSTAT-CHF. *Eur J Heart Fail*. 2016;18:716-726.

S7. Pires Correia C, Neves I, Chaves P, Cardoso T, Silva R, Almeida J. Minimal Change Disease and Phenylketonuria in an Adult Patient: The Two Sides of Protein Homeostasis. *Eur J Case Rep Intern Med*. 2020;7:001821.
