# Supplementary material for: Large Variations in Phenylalanine Concentrations Associate Adverse Cardiac Remodelling in Adult Patients With Phenylketonuria—A Long‐Term CMR Study
Source: J Cachexia Sarcopenia Muscle. 2025 Jan 10;16(1):e13667. doi: 10.1002/jcsm.13667 (PMC11724155; doi:10.1002/jcsm.13667)
Supplement: Supplementary file 2 — Table S1. CMR Parameters: Volumes, Parametric Imging – in PKU patients at follow‐up and a matched Control group. Table S 2. CMR parameters: strain, haemodynamic forces in PKU patients at follow‐up and a matched Control group. Table S3. Influence of phenylalanine variation on cardiovascular phenotype in PKU patients – univariate (left columns) and co‐variate with BMI and age (right columns). Table S4. Influence of phenylalanine variation on cardiovascular phenotype in PKU patients – significance of interaction between patient sex and phenylalanine variation and cardiac parameters (left), univariate linear regression in the two subgroups of follow‐up PKU patients, males and females (right). [file JCSM-16-e13667-s002.docx]

|  | PKU patients (N=15) | Control  (N=20) | P Value |
| --- | --- | --- | --- |
|  |  |  |  |
| Age, y | 39.8±8.1 | 40.3±6.6 | 0.84 |
| Males, n (%) | 9(60) | 11(55) | 0.77 |
| Height, cm | 173±11 | 172±10 | 0.78 |
| Weight, kg | 84±20 | 75±15 | 0.14 |
| BMI, kg/m² | 28.7±6.3 | 25.4±3.1 | ***0.049*** |
| BSA, m² | 2.00±0.26 | 1.89±0.22 | 0.18 |
| **Left Ventricle** |  |  |  |
| LV EDVi, mL/m² | 72±13 | 78±12 | 0.17 |
| LV ESVi, mL/m² | 31±9 | 29±7 | 0.46 |
| LV SVi, mL/m² | 41±7 | 47±6 | ***0.010*** |
| LV EF, % | 57±7 | 62±5 | ***0.019*** |
| LV CI, L/min/mL/m² | 3.0±0.5 | 2.8±0.5 | 0.25 |
| Septal ED WT, mm | 7.2±1.8 | 8.1±1.6 | 0.13 |
| Lateral ED WT, mm | 6.2±1.5 | 7.7±1.1 | ***0.002*** |
| Relative WT | 1.2±0.1 | 1.1±0.2 | 0.09 |
| LVMi, g/m² | 40±10 | 50±10 | ***0.019*** |
| LVM/EDV, g/mL | 0.58±0.23 | 0.54±0.15 | 0.54 |
| LV ED maximal diameter, mm | 53±5 | 44±4 | ***<0.001*** |
| **Left Atrium** |  |  |  |
| LAVmax index, mL/m² | 38±7 | 40±7 | 0.41 |
| LAVmin index, mL/m² | 14±5 | 15±3 | 0.47 |
| LA emptying fraction % | 68±6 | 63±4 | ***0.006*** |
| **Right Ventricle** |  |  |  |
| RV EDVi, mL/m² | 78±17 | 75±17 | 0.61 |
| RV ESVi, mL/m² | 39±11 | 32±9 | ***0.046*** |
| RV SVi, mL/m² | 40±8 | 43±10 | 0.35 |
| RV ejection fraction, % | 51±4 | 58±5 | ***<0.001*** |
| RV CI, mL/m² | 2.9±0.5 | 2.6±0.7 | 0.17 |
| **Ascending Aorta** |  |  |  |
| Systolic aortic area, cm2 | 4.53±0.92 | 4.47±0.85 | 0.84 |
| Diastolic aortic area, cm2 | 3.72±0.87 | 3.70±0.79 | 0.94 |
| Ao distens, 10-3mmHg-1 | 5.21±1.17 | 5.26±1.03 | 0.89 |
| **Parametric Imaging** |  |  |  |
| T1 native, ms | 1010±35 | 972±29 | ***0.001*** |
| ECV (%) | 26.5±3.7 | 25.1±4.2 | 0.31 |
| T2, ms | 48.9±3.1 | 48.1±2.0 | 0.36 |

Supplemental Table 1. CMR Parameters: Volumes, Parametric Imging – in PKU patients at follow-up and a matched Control group

Abbreviations: LV left ventricle, EDVi indexed end-diastolic volume, ESVi indexed end-systolic volume, ED end-diastolic, ES end-systolic, CI cardiac index, WT wall thickness, LVM left ventricular mass, LA left atrium, RV right ventricle, ECV extracellular volume.

|  | PKU patients (N=15) | Control  (N=20) | P Value |
| --- | --- | --- | --- |
|  |  |  |  |
| **Left Ventricle** |  |  |  |
| GLS Endo, % | -26.9±5.8 | -25.9±3.1 | 0.52 |
| GLS Myo, % | -24.9±3.1 | -24.1±2.3 | 0.39 |
| GLS Endo-Epi Gradient, % | -4.3±6.0 | -4.2±2.4 | 0.95 |
| GCS Endo, % | -31.1±5.2 | -35.7±5.7 | ***0.020*** |
| GCS Myo, % | -20.7±2.5 | -23.8±3.4 | ***0.005*** |
| GCS Endo-Epi Gradient, % | -16.9±5.5 | -19.7±5.0 | 0.13 |
| HD Syst Force (%) | 33.4±8.0 | 35.0±11.4 | 0.65 |
| HD Syst Work (%) | 5.0±2.4 | 6.6±3.5 | 0.14 |
| HD Syst Work (mJ) | 4.1±1.5 | 5.0±2.1 | 0.17 |
| HD Syst Power (mJ/s) | 48.8±20.6 | 45.4±19.7 | 0.62 |
| HD Early Diast Force (%) | -26.5±12.2 | -11.2±5.5 | ***<0.001*** |
| HD Early Diast Work (%) | -1.4±1.0 | -0.9±0.4 | ***0.049*** |
| HD Early Diast Work (mJ) | -1.2±0.8 | -0.6±0.3 | ***0.004*** |
| HD Early Diast Power (mJ/s) | -20.1±13.5 | -8.2±4.2 | ***<0.001*** |
| HD Late Diast Force (%) | -12.1±6.2 | -9.0±4.5 | 0.10 |
| **Left Atrium** |  |  |  |
| LA strain, % | 37.3±9.6 | 33.1±7.4 | 0.15 |
| **Right Ventricle** |  |  |  |
| RV longitudinal strain, % | -34.1±5.2 | -32.3±5.4 | 0.33 |

Supplemental Table 2. CMR Parameters: Strain, Haemodynamic Forces in PKU patients at follow-up and a matched Control group

Abbreviations: GLS global longitudinal strain, GCS global circumferential strain, Endo subendocardial, Myo mid-myocardium, Epi subepicardial layers, HD hemodynamic, Syst systolic, Diast diastolic, LV left ventricle, LA left atrium, RV right ventricle

|  | Phenylalanine Gradient | | Phenylalanine Gradient controlled for age and BMI | |
| --- | --- | --- | --- | --- |
|  | β | P Value | β | P Value |
|  |  |  | * controlled for age only |  |
| **Anthropometrics** |  |  |  |  |
| Δ Weight, kg | 0.56 | ***0.031*** | 0.53* | ***0.045*** |
| Δ BMI, kg/m2 | 0.55 | ***0.035*** | 0.53* | ***0.049*** |
| Δ BSA, m2 | 0.58 | ***0.023*** | 0.56* | ***0.034*** |
| **Left Ventricle** |  |  |  |  |
| Δ LV EDV, mL | 0.11 | 0.69 |  |  |
| Δ LV ESV, mL | 0.51 | ***0.050*** | 0.53 | ***0.044*** |
| Δ LV SV, mL | 0.34 | 0.22 |  |  |
| Δ LV EF, % | 0.61 | ***0.017*** | 0.56 | ***0.012*** |
| Δ LV CO, L/min | 0.28 | 0.31 |  |  |
| Δ Septal ED WT, mm | -0.42 | 0.12 |  |  |
| Δ Lateral ED WT, mm | -0.35 | 0.38 |  |  |
| Δ LV Mass (g) | -0.15 | 0.59 |  |  |
| Δ LV Mass/ED volume, g/mL | -0.06 | 0.83 |  |  |
| Δ GLS Endo, % | -0.63 | ***0.012*** | -0.63 | ***0.011*** |
| Δ GCS Endo, % | -0.28 | 0.32 |  |  |
| Δ HD Syst Force (%) | -0.17 | 0.54 |  |  |
| Δ HD Syst Work (%) | 0.01 | 0.98 |  |  |
| Δ HD Syst Work (mJ) | -0.12 | 0.66 |  |  |
| Δ HD Syst Power (mJ/s) | -0.19 | 0.50 |  |  |
| Δ HD Early Diast Force (%) | -0.06 | 0.82 |  |  |
| Δ HD Early Diast Work (%) | 0.06 | 0.85 |  |  |
| Δ HD Early Diast Work (mJ) | 0.23 | 0.40 |  |  |
| Δ HD Early Diast Power (mJ/s) | 0.4 | 0.14 |  |  |
| Δ HD Late Diast Force (%) | 0.08 | 0.78 |  |  |
| **Left Atrium** |  |  |  |  |
| Δ LA max Vol, mL | -0.24 | 0.39 |  |  |
| Δ LA emptying fraction % | 0.75 | ***0.001*** | 0.77 | ***0.003*** |
| Δ LA strain, % | 0.51 | 0.05 |  |  |
| Right Ventricle |  |  |  |  |
| Δ RV EDV, mL | 0.27 | 0.33 |  |  |
| Δ RV ESV, mL | 0.1 | 0.73 |  |  |
| Δ RV SV, mL | 0.27 | 0.34 |  |  |
| Δ RV EF, % | 0.21 | 0.46 |  |  |
| Δ RV CO , L/min | 0.2 | 0.47 |  |  |
| Δ RV GLS, % | -0.17 | 0.55 |  |  |
| **Ascending Aorta** |  |  |  |  |
| Δ Ao distens, 10-3mmHg-1 | -0.11 | 0.70 |  |  |
| **Parametric Imaging** |  |  |  |  |
| Δ T1 native, ms | -0.78 | ***<0.001*** | -0.77 | ***0.002*** |
| Δ ECV, % | -0.61 | ***0.016*** | -0.61 | ***0.014*** |

Supplemental Table 3. Influence of phenylalanine variation on cardiovascular phenotype in PKU patients – univariate (left columns) and co-variate with BMI and age (right columns).

Abbreviations:BMI body mass index, BSA body surface area, LV left ventricle, EDV end-diastolic volume, ESV end-systolic volume, SV stroke volume, CO cardiac output, WT wall thickness,, GLS global longitudinal strain, GCS global circumferential strain, Endo subendocardial, HD hemodynamic, Syst systolic, Diast diastolic, LA left atrium, RV right ventricle, Ao disten aortic distensibility, ECV extracellular volume. As ΔPhe is correlated to estimated ΔBSA, measured and not indexed values of CMR parameters were included in this analysis. As BMI is correlated with Weight and BSA, the variations in Wight, BMI and BSA were covariate with age only (*).

|  | GLM - Interaction M-F vs Parameter | Linear Regression vs Phenylalanine Gradient | | | |
| --- | --- | --- | --- | --- | --- |
|  |  | Males | | Females | |
|  | P Value | β | P Value | β | P Value |
| **Anthropometrics** |  |  |  |  |  |
| Δ Weight, kg | ***0.036*** | 0.68 | ***0.044*** | 0.19 | 0.72 |
| Δ BMI, kg/m2 | ***0.049*** | 0.65 | 0.06 | 0.35 | 0.50 |
| Δ BSA, m2 | ***0.015*** | 0.71 | ***0.024*** | 0.12 | 0.82 |
| **Left Ventricle** |  |  |  |  |  |
| Δ LV EDV, mL | 0.25 |  |  |  |  |
| Δ LV ESV, mL | 0.07 |  |  |  |  |
| Δ LV SV, mL | 0.20 |  |  |  |  |
| Δ LV EF, % | ***0.050*** | 0.83 | ***0.005*** | -0.05 | 0.92 |
| Δ LV CO, L/min | 0.38 |  |  |  |  |
| Δ Septal ED WT, mm | ***0.023*** | -0.73 | ***0.024*** | 0.04 | 0.94 |
| Δ Lateral ED WT, mm | 0.07 |  |  |  |  |
| Δ LV Mass (g) | 0.42 |  |  |  |  |
| Δ LV Mass/ED volume, g/mL | 0.89 |  |  |  |  |
| Δ GLS Endo, % | ***0.049*** | -0.75 | ***0.020*** | -0.54 | 0.27 |
| Δ GCS Endo, % | 0.42 |  |  |  |  |
| Δ HD Syst Force (%) | 0.78 |  |  |  |  |
| Δ HD Syst Work (%) | 0.99 |  |  |  |  |
| Δ HD Syst Work (mJ) | 0.81 |  |  |  |  |
| Δ HD Syst Power (mJ/s) | 0.73 |  |  |  |  |
| Δ HD Early Diast Force (%) | 0.67 |  |  |  |  |
| Δ HD Early Diast Work (%) | 0.69 |  |  |  |  |
| Δ HD Early Diast Work (mJ) | 0.14 |  |  |  |  |
| Δ HD Early Diast Power (mJ/s) | 0.09 |  |  |  |  |
| Δ HD Late Diast Force (%) | 0.50 |  |  |  |  |
| **Left Atrium** |  |  |  |  |  |
| Δ LA max Vol, mL | 0.34 |  |  |  |  |
| Δ LA emptying fraction % | ***0.004*** | 0.79 | ***0.011*** | 0.81 | ***0.049*** |
| Δ LA strain, % | ***0.036*** | 0.25 | 0.52 | 0.82 | ***0.047*** |
| **Right Ventricle** |  |  |  |  |  |
| Δ RV EDV, mL | 0.41 |  |  |  |  |
| Δ RV ESV, mL | 0.94 |  |  |  |  |
| Δ RV SV, mL | 0.33 |  |  |  |  |
| Δ RV EF, % | 0.51 |  |  |  |  |
| Δ RV CO , L/min | 0.50 |  |  |  |  |
| Δ RV GLS, % | 0.11 |  |  |  |  |
| **Ascending Aorta** |  |  |  |  |  |
| Δ Ao distens, 10-3mmHg-1 | 0.84 |  |  |  |  |
| **Parametric Imaging** |  |  |  |  |  |
| Δ T1 native, ms | ***<0.001*** | -0.94 | ***<0.001*** | -0.63 | 0.18 |
| Δ ECV, % | 0.06 |  |  |  |  |
| Δ T2, ms | 0.55 |  |  |  |  |

Supplemental Table 4. Influence of phenylalanine variation on cardiovascular phenotype in PKU patients – significance of interaction between patient sex and phenylalanine variation and cardiac parameters (left), univariate linear regression in the two subgroups of follow-up PKU patients, males and females (right).

Abbreviations:GLM generalized linear model, BMI body mass index, BSA body surface area, LV left ventricle, EDV end-diastolic volume, ESV end-systolic volume, SV stroke volume, CO cardiac output, WT wall thickness,, GLS global longitudinal strain, GCS global circumferential strain, Endo subendocardial, HD hemodynamic, Syst systolic, Diast diastolic, LA left atrium, RV right ventricle, Ao disten aortic distensibility, ECV extracellular volume. As ΔPhe is correlated to estimated ΔBSA, measured and not indexed values of CMR parameters were included in this analysis
